# Supplementary material for: Near Chromosome-Level Genome Assembly and Annotation of Rhodotorula babjevae Strains Reveals High Intraspecific Divergence
Source: J Fungi (Basel). 2022 Mar 22;8(4):323. doi: 10.3390/jof8040323 (PMC9027234; doi:10.3390/jof8040323)
Supplement: Supplementary file 1 [file jof-08-00323-s001.zip › jof-1504618-supplementary.pdf]

Supplementary information

Near chromosome-level genome assembly and annotation of *Rhodotorula babjevae* strains reveals high intraspecific divergence

Giselle C. Martín-Hernández[1], Bettina Müller[1], Christian Brandt[2], Martin Hölzer[3], Adrian Viehweger[4], Volkmar Passoth[1,*]

[1]Department of Molecular Sciences, Swedish University of Agricultural Sciences, 75007 Uppsala, Sweden

[2]Institute for Infectious Diseases and Infection Control, Jena University Hospital, Jena, Germany

[3]Method Development and Research Infrastructure, MF1 Bioinformatics, Robert Koch Institute, Berlin, Germany

[4]Institute of Medical Microbiology and Virology, University Hospital Leipzig, Germany

[*]Author for Correspondence: Volkmar Passoth, Department of Molecular Sciences, Swedish University of Agricultural Sciences, Uppsala, Sweden, +4618673380, Volkmar.Passoth@slu.se

G.C.M.-H. and B.M. equally contributed to this paper and shall both be regarded as first authors

Supplementary Table S1. Program versions used for the genome assembly and annotation pipeline

Supplementary Table S2. Characteristics from the contigs and scaffolds of *Rhodotorula babjevae* CBS 7808 genome assembly

Supplementary Table S3. Characteristics from the contigs and scaffolds of *Rhodotorula babjevae* DBVPG 8058 genome assembly

Supplementary Table S4. Distribution of exon counts in the two strains of *Rhodotorula babjevae*.

Supplementary Table S5. Examples of lipid and carotenoid metabolism related genes in *Rhodotorula babjevae* CBS 7808

Supplementary Table S6. Examples of lipid and carotenoid metabolism related genes in *Rhodotorula babjevae* DBVPG 8058

Supplementary Table S7. Scaffold and contigs with assigned homology and pairwise nucleotide identity between *Rhodotorula babjevae* strains

Supplementary Table S8. Summary of features of strain-unique contigs in *Rhodotorula babjevae*

Supplementary Table S9. Genetic divergence between *Rhodotorula babjevae* strains and closely related *Rhodotorula* species

Supplementary Table S10. Alignment statistics of the duplicated genes identified in the *Rhodotorula babjevae* CBS 7808 genome by OrthoVenn2

Supplementary Table S11. Alignment statistics of the duplicated genes identified in the *Rhodotorula babjevae* DBVPG 8058 genome by OrthoVenn2

Supplementary Table S12. Alignment statistics of the duplicated genes identified in the *Rhodotorula babjevae* CBS 7808 genome by BLASTp

Supplementary Table S13. Alignment statistics of the duplicated genes identified in the *Rhodotorula babjevae* DBVPG 8058 genome by BLASTp

Supplementary Table S14. Duplicated genes in *Rhodotorula babjevae* identified by BLASTp and OrthoVenn2 with a minimum coverage of 70%

Supplementary Figure S1. Gene Ontology (GO) term summary related to the GO topic: molecular functions

Supplementary Figure S2. Gene Ontology (GO) term summaries belonging to the GO topic: biological processes

Supplementary Figure S3. Gene Ontology (GO) term summaries belonging to the GO topic: cellular components

Supplementary Figure S4. Examples of lipid metabolism pathways in *Rhodotorula babjevae* CBS 7808 reconstructed by KEGG Mapper

Supplementary Figure S5. Examples of lipid metabolism pathways in *Rhodotorula babjevae* DBVPG 8058 reconstructed by KEGG Mapper

Supplementary Figure S6. Quantitative assessment of the hybrid genome assemblies and annotation completeness using Benchmarking Universal Single-Copy Orthologs (BUSCO)

Supplementary Figure S7. LASTZ alignment of the mitochondrial genome sequences of *Rhodotorula babjevae* CBS 7808 and DBVPG 8058

Supplementary Figure S8. LASTZ alignment of contigs with assigned homology of *Rhodotorula babjevae* CBS 7808 and DBVPG 8058 representing putative chromosomes

Supplementary Table S1. Program versions used for the genome assembly and annotation pipeline

Program	Version
Bandage	0.8.1
BBMap	38.86
Biopython	1.74
BioRuby	2.0.1
BUSCO	3.0.2
BWA	0.7.17
chromoMap	0.2
dammit	1.2
fastp	0.20.0
FastQC	0.11.8
Filtlong	0.2.0
Flye	2.8
ggplot2	3.3.1
HISAT	2.2.0
Medaka	0.10.0
MetaEuk	1.ea903e5
minimap2	2.17
MITOS	2.0.3
MultiQC	1.8
NanoPlot	1.25.0
Pilon	1.23
Racon	1.4.7
RepeatMasker	4.0.9
samtools	1.10
simple-circularise	1.0
SortMeRNA	2.1b
sourmash	2.0.1
StringTie	2.1.1
Tapestry	1.0.0

Supplementary Table S2. Characteristics from the contigs and scaffolds of *Rhodotorula babjevae* CBS 7808 genome assembly

**Contig/scaffold**	**Length (bp)**	**GC content (%)**	**Read depth**	**No. of telomeric regions**
ontig 10	1,067,634	67.97	39	0
contig 11	1,300,441	68.18	42	1
contig 12	511,897	68.45	33	0
contig 2	30,876	38.85	892	2
contig 25	627,118	68.03	47	0
contig 26	40,644	57.95	20	1
contig 27	320,063	67.64	31	1
contig 28	422,133	67.84	36	0
contig 3	1,574,520	68.39	49	1
contig 30	1,569,459	68.66	48	0
contig 31	979,228	68.28	40	1
contig 36	1,056,323	68.50	52	0
contig 37	362,52	68.05	34	0
contig 38	881,966	68.53	51	1
contig 39	564,129	68.13	41	1
contig 4	418,972	67.71	38	1
contig 45	762,86	68.32	37	1
contig 46	8,677	65.90	79	0
contig 5	2,415,752	68.54	46	1
contig 62	644,441	68.12	42	0
contig 65	630,535	68.27	52	1
contig 66	406,102	68.73	45	0
contig 7	1,460,653	68.35	46	1
contig 9	429,397	68.87	36	1
scaffold 40	948,604	68.04	50	0
scaffold 49	1,089,446	67.93	52	0
scaffold 6	1,337,997	68.23	46	1

Supplementary Table S3. Characteristics from the contigs and scaffolds of *Rhodotorula babjevae* DBVPG 8058 genome assembly

Contig/scaffold	Length (bp)	GC content (%)	Read depth	No. of telomeric regions
contig 1	565,532	67.52	44	1
contig 20	637,402	68.57	43	0
contig 33	529,001	68.19	42	2
contig 38	1,780,658	68.22	43	0
contig 40	1,004,683	68.30	43	1
contig 42	1,446,680	68.41	45	0
contig 44	357,974	68.15	44	0
contig 45	394,205	68.00	42	0
contig 46	670,828	68.68	42	1
contig 47	557,103	68.12	43	0
contig 48	931,129	68.59	43	1
contig 49	396,114	68.44	40	1
contig 51	924,743	68.52	45	1
contig 53	571,073	67.98	43	1
contig 54	766,724	68.39	40	0
contig 57	1,049,892	68.40	43	1
contig 65	41,334	67.14	59	1
contig 66	419,035	67.94	44	1
contig 68	408,627	68.12	39	0
contig 69	1,447,990	68.48	45	1
contig 70	789,767	68.03	43	0
contig 71	449,691	68.69	42	1
contig 72	5,97	48.69	306	2
contig 73	659,761	67.77	41	0
contig 74	614,034	68.46	41	0
contig 75	900,917	68.16	43	0
contig 77	446,828	68.09	43	1
contig 79	28,432	38.89	1160	1
contig 82	299,18	68.75	40	2
contig 83	6,381	59.36	101	0
contig 84	425,34	67.39	45	0
contig 85	573,802	68.36	41	1
contig 86	443,617	68.68	42	1
scaffold 52	977,625	68.29	43	1

Supplementary Table S4. Distribution of exon counts in the two strains of *Rhodotorula babjevae*.

Strain	CBS 7808	DBVPG 8058
Exons per transcript	Number of transcripts	
1	1201	1176
2	1400	1417
3	1367	1366
4	1108	1154
5	808	767
6	581	539
7	391	385
8	275	268
9	168	159
10	121	95
11	61	68
12	42	54
13	36	29
14	19	17
15	11	8
16	5	5
17	4	3
18	2	2
19	2	1
20	3	1
21	0	1
23	0	1
24	2	0
Total	7607	7516

Supplementary Table S5. Examples of lipid and carotenoid metabolism related genes in *Rhodotorula babjevae* CBS 7808

Gene	Enzyme	EC	Pathway	Contig/scaffold	Gene ID
CDC19	Pyruvate kinase 1	2.7.1.40	Glycolysis	contig 10	NANOZOOG247, NANOZOOG248
MAE1	NADP-dependent malic enzyme	1.1.1.40	NADPH regeneration	contig 10	NANOZOOG21
MAE2	NAD-dependent malic enzyme 3	1.1.1.38	Tricarboxylic acid cycle	contig 36	NANOZOOG2566
ACL2	ATP-citrate synthase subunit 2	2.3.3.8	Lipid synthesis	contig 30	NANOZOOG1597
ACL1	ATP-citrate synthase subunit 1	2.3.3.8	Lipid synthesis	contig 30	NANOZOOG1598
ACC1	Acetyl-CoA carboxylase	6.4.1.2	Fatty acid synthesis	scaffold 6	NANOZOOG7298, NANOZOOG7299, NANOZOOG7300
FAS1	β- subunit of fatty acid synthase complex	2.3.1.86	Fatty acid synthesis	scaffold 6	NANOZOOG7332, NANOZOOG7509
FAS2	α- subunit of fatty acid synthase complex	2.3.1.86	Fatty acid synthesis	scaffold 6	NANOZOOG7507, NANOZOOG7508
OLE1	Acyl-CoA desaturase	1.14.19.1	Unsaturated fatty acid synthesis	contig 30	NANOZOOG1596
ACAD11	Acyl-CoA dehydrogenase family member 11	1.3.99	Fatty acid beta-oxidation	scaffold 6	NANOZOOG7232
IBR3	Probable acyl-CoA dehydrogenase IBR3	1.3.99	Fatty acid beta-oxidation	scaffold 49	NANOZOOG6894
ECI1	Enoyl-CoA hydratase 1	4.2.1.17	Fatty acid beta-oxidation	scaffold 49	NANOZOOG6861
ECI1	Enoyl-CoA hydratase 1	4.2.1.17	Fatty acid beta-oxidation	contig 11	NANOZOOG791
POT1	Acetyl-CoA acyltransferase	2.3.1.16	Fatty acid beta-oxidation	contig 3	NANOZOOG3582, NANOZOOG3634
POT1	Acetyl-CoA acyltransferase	2.3.1.16	Fatty acid beta-oxidation	contig 65	NANOZOOG5517
POT1	Acetyl-CoA acyltransferase	2.3.1.16	Fatty acid beta-oxidation	contig 36	NANOZOOG2634
LRO1	Phospholipid:diacylglycerol acyltransferase	2.3.1.158	Glicerolipid synthesis	contig 5	NANOZOOG4705
HMG1	HMG-CoA reductase	1.1.1.34	Isoprenoid synthesis	contig 31	NANOZOOG2214, NANOZOOG2215, NANOZOOG2216
HCS1	HMG-CoA synthase	2.3.3.10	Isoprenoid synthesis	contig 62	NANOZOOG5192
ERG8	Phosphomevalonate kinase, peroxisomal	2.7.4.2	Isoprenoid synthesis	scaffold 6	NANOZOOG7354
crtYB	Bifunctional lycopene cyclase/phytoene synthase	5.5.1.19 & 2.5.1.32	Carotenoid synthesis	scaffold 6	NANOZOOG7200
crtI	Phytoene desaturase	1.3.99.30	Carotenoid synthesis	scaffold 6	NANOZOOG7203
BTS1	Geranylgeranyl pyrophosphate synthase	2.5.1.-	Carotenoid synthesis	contig 5	NANOZOOG4762

Supplementary Table S6. Examples of lipid and carotenoid metabolism related genes in *Rhodotorula babjevae* DBVPG 8058

Gene	Enzyme	EC	Pathway	Contig/scaffold	Gene ID
CDC19	Pyruvate kinase 1	2.7.1.40	Glycolysis	contig 54	NANOZOOG3961, NANOZOOG3962
MAE1	NADP-dependent malic enzyme	1.1.1.40	NADPH regeneration	contig 47	NANOZOOG2569
MAE2	NAD-dependent malic enzyme 2	1.1.1.38	Tricarboxylic acid cycle	contig 57	NANOZOOG4100
ACL1	ATP-citrate synthase subunit 1	2.3.3.8	Lipid synthesis	contig 20	NANOZOOG209
ACC1	Acetyl-CoA carboxylase	6.4.1.2	Fatty acid synthesis	contig 70	NANOZOOG5315, NANOZOOG5316, NANOZOOG5317, NANOZOOG5318
FAS1	β- subunit of fatty acid synthase complex	2.3.1.86	Fatty acid synthesis	contig 70	NANOZOOG5349
FAS2	α- subunit of fatty acid synthase complex	2.3.1.86	Fatty acid synthesis	contig 33	NANOZOOG458, NANOZOOG459
OLE1	Acyl-CoA desaturase	1.14.19.1	Unsaturated fatty acid synthesis	contig 20	NANOZOOG210
ACAD10	Acyl-CoA dehydrogenase family member 10	1.3.99	Fatty acid beta-oxidation	contig 53	NANOZOOG3588
ACAD11	Acyl-CoA dehydrogenase family member 11	1.3.99	Fatty acid beta-oxidation	contig 70	NANOZOOG5252
IBR3	Probable acyl-CoA dehydrogenase IBR3	1.3.99	Fatty acid beta-oxidation	contig 40	NANOZOOG1380
D6C81 05617	Enoyl-CoA hydratase 1	4.2.1.17	Fatty acid beta-oxidation	contig 40	NANOZOOG1413
POT1	Acetyl-CoA acyltransferase	2.3.1.16	Fatty acid beta-oxidation	contig 57	NANOZOOG4029
POT1	Acetyl-CoA acyltransferase	2.3.1.16	Fatty acid beta-oxidation	contig 74	NANOZOOG5938
POT1	Acetyl-CoA acyltransferase	2.3.1.16	Fatty acid beta-oxidation	contig 75	NANOZOOG6026, NANOZOOG6080
HMG1	HMG-CoA reductase	1.1.1.34	Isoprenoid synthesis	contig 82	NANOZOOG6558, NANOZOOG6559, NANOZOOG6560
HCS1	HMG-CoA synthase	2.3.3.10	Isoprenoid synthesis	contig 38	NANOZOOG1015
crtYB	Bifunctional lycopene cyclase/phytoene synthase	5.5.1.19 & 2.5.1.32	Carotenoid synthesis	contig 70	NANOZOOG5230
crtI	Phytoene desaturase	1.3.99.30	Carotenoid synthesis	contig 70	NANOZOOG5227
BTS1	Geranylgeranyl pyrophosphate synthase	2.5.1.-	Carotenoid synthesis	contig 69	NANOZOOG5016

Supplementary Table S7. Scaffold and contigs with assigned homology and pairwise nucleotide identity between *Rhodotorula babjevae* strains (deduced from LASTZ alignment, Figure S8).

DBVPG 8058	CBS 7808																										
	2	3	4	5	7	9	10	11	12	25	26	27	28	30	31	36	37	38	39	45	46	62	65	66	6	40	49
1																			85								
20														84													
33																									82		
38												85						85				85					
40																											83
42								84						84													
44								86						64													
45			82																								
46		85																									
47							82																				
48					84																						
49																								85			
51																										84	
53					86																						
54							85													85							
57																83											
65																											91
66													84														
68									84								84										
69				84																							
70																									83		
71									86								86										
72																											
73															84												
74																							83				
75		84																									
77																				86							
79	97																										
82															85												
83																											
84								85																			
85										85																	
86						87																					
52				84																							

The contigs corresponding to the mitochondrial genome are denoted in yellow and to putative extrachromosomal elements in green.

Supplementary Table S8. Summary of features from strain-unique contigs in *Rhodotorula babjevae*

Contig name	*R. babjevae* strain number	Size (bp)	GC content (%)	Relative read depth*	Size of circular contig (bp)
contig 26	CBS 7808	40,644	57.95	0,5	30,109
contig 46	CBS 7808	8,677	65.9	1,8	-
contig 83	DBVPG 8058	6,381	59.36	2,4	-
contig 72	DBVPG 8058	5,97	48.69	7,2	-

*Read depth of the contig divided by the average read depth of contigs with assigned homology between *Rhodotorula babjevae* strains

Supplementary Table S9. Genetic divergence between *Rhodotorula babjevae* strains and closely related *Rhodotorula* species

			Rhodotorula babjevae CBS 7808			
		DNA-DNA homology		ANI	*kr*	Accesion number
Strain	Distance	DDH estimate	Difference in % G+C			
R. babjevae DBVPG 8058	0.5787	44.20%	0.00	84.48%	0.088959	this study
R. graminis WP1	0.6415	39.00%	0.47	82.85%	0.098387	JTAO00000000.1
R. glutinis ZHK	0.6240	40.40%	0.43	83.43%	0.095288	JAAGPT000000000.1
R. toruloides CBS 14	0.9350	22.40%	6.40	77.01%	0.221540	PRJEB40807
			Rhodotorula babjevae DBVPG 8058			
R. babjevae CBS 7808	0.5787	44.20%	0.00	84.48%	0.088959	this study
R. graminis WP1	0.6349	39.50%	0.48	82.88%	0.096059	JTAO00000000.1
R. glutinis ZHK	0.6233	40.40%	0.43	83.26%	0.095886	JAAGPT000000000.1
R. toruloides CBS 14	0.9319	22.50%	6.40	77.36%	0.205772	PRJEB40807
			Rhodotorula toruloides CBS 14			
R. toruloides NP11	0.0893	97.60%	0.21	100%	0.000445	ALAU00000000.1
R. toruloides CGMCC 2.1609	0.3725	66.80%	0.10	99.27%	0.000337	LKER00000000.1
R. toruloides VN1	0.6319	39.7%	0.07	85.48%	0.094063	SJTE00000000.1
R. toruloides NBRC 0880	0.6456	38.60%	0.00	85.15%	0.101898	LCTV00000000.2

ANI, Average Nucleotide Identity values using the web-based calculator available at Kostas Lab [17]; DDH, DNA–DNA homology estimated using the Genome-to-Genome Distance Calculator 2.1 with GBDP2 MUMMER program [18].

Supplementary Table S10. Alignment statistics of the duplicated genes identified in the *Rhodotorula babjevae* CBS 7808 genome by OrthoVenn2

Contig/ scaffold	Query	Subject	Inflation	Query start	Query stop	Subject start	Subject stop	% identity	% coverage	Mismatches	Gaps	Gap Bases	Score	E-Value	Gene ID	Strand
contig 10	7	2	1,1	1	218	5	222	100	46	0	0	0	1135	7E-154	NANOZOOG8	-
				290	456	222	385	95	34	6	1	3	783	1E-100		
contig 10	2	7	1,1	5	222	1	218	100	55	0	0	0	1135	6E-154	NANOZOOG3	+
				222	385	290	456	95	41	6	1	3	783	9E-101		
contig 10	84	701	0,9	12	359	1	401	68	94	66	7	63	1338	0E+00	NANOZOOG85	-
contig 11	701	84	0,9	1	401	12	359	68	84	66	7	63	1338	0E+00	NANOZOOG701	-
contig 12	801	812	0,8	1	280	1	304	89	82	8	1	24	1379	0E+00	NANOZOOG801	+
contig 12	812	801	0,8	1	304	1	280	89	91	8	1	24	1379	0E+00	NANOZOOG812	-
contig 12	830	831	0,5	13	178	1	166	100	93	0	0	0	894	1E-125	NANOZOOG830	+
contig 12	831	830	0,5	1	166	13	178	100	97	0	0	0	894	1E-125	NANOZOOG831	-
contig 12	865	866	0,7	278	663	194	574	47	45	82	9	167	885	4E-111	NANOZOOG865	+
contig 12	866	865	0,7	194	574	278	663	47	52	82	9	167	891	5E-112	NANOZOOG866	+
				1	92	118	236	56	16	25	3	27	363	6E-37		
contig 27	1195	1201	1,8	1144	1928	18	755	91	38	24	4	47	3062	0E+00	NANOZOOG1195	-
contig 27	1201	1195	1,8	18	755	1144	1928	91	90	24	4	47	3065	0E+00	NANOZOOG1201	+
contig 10	122	123	0,4	8	190	1	211	84	91	5	1	28	873	2E-121	NANOZOOG123	+
contig 10	123	122	0,4	1	211	8	190	87	87	0	1	28	905	3E-126	NANOZOOG124	-
contig 28	1319	1324	0,7	7	308	256	553	76	96	68	3	4	1123	2E-152	NANOZOOG1319	-
contig 28	1324	1319	0,7	1	245	62	309	83	44	38	2	3	960	1E-127	NANOZOOG1324	+
contig 30	1409	1960	1,8	1	789	26	830	90	58	68	2	16	3512	0E+00	NANOZOOG1409	-
contig 30	1960	1409	1,8	26	830	1	789	90	95	63	2	16	3419	0E+00	NANOZOOG1956	+
contig 30	1409	2182	1,8	1	1354	3	1301	87	96	112	5	59	5497	0E+00	NANOZOOG1409	-
contig 31	2182	1409	1,8	3	1301	1	1354	87	99	111	5	59	5492	0E+00	NANOZOOG2177	+
contig 30	1409	5744	1,8	1	1354	3	1301	87	96	112	5	59	5497	0E+00	NANOZOOG1409	-
contig 66	5744	1409	1,8	3	1340	1	1354	90	100	112	4	20	5706	0E+00	NANOZOOG5732	+
contig 30	1428	1430	1,0	1	294	1	290	99	58	0	1	4	1501	0E+00	NANOZOOG1428	-
contig 30	1430	1428	1,0	154	290	1	137	100	47	0	0	0	701	5E-92	NANOZOOG1429	+
contig 30	1500	1501	0,7	15	276	30	296	76	94	55	4	9	1038	9E-144	NANOZOOG1499	-
contig 30	1501	1500	0,7	1	301	1	301	100	100	0	0	0	1566	0E+00	NANOZOOG1500	-
contig 30	1515	1516	0,6	1	189	31	219	100	99	0	0	0	983	3E-138	NANOZOOG1514	+
contig 30	1516	1515	0,6	31	219	1	189	100	86	0	0	0	983	3E-138	NANOZOOG1515	-
contig 30	1587	1588	0,2	1	204	1	242	84	100	0	1	38	782	4E-103	NANOZOOG1586	-
contig 30	1588	1587	0,2	1	242	1	204	84	38	0	1	38	782	1E-102	NANOZOOG1587	+
contig 30	1749	1763	1,0	1	509	1	551	64	89	80	13	134	1713	0E+00	NANOZOOG1746	+
contig 30	1763	1749	1,0	1	551	1	509	64	84	80	13	134	1713	0E+00	NANOZOOG1760	-
contig 30	1886	5661	0,5	140	358	120	337	73	59	47	4	15	702	4E-91	NANOZOOG1883	-
				1	27	74	100	96	8	1	0	0	133	3E-09		
contig 66	5661	1886	0,5	122	337	142	358	75	64	51	3	3	767	6E-101	NANOZOOG5649	-

				74	100	1	27	96	8	1	0	0	134	3E-09		
contig 30	1917	1931	0,7	67	400	170	511	64	82	105	4	22	1137	1E-153	NANOZOOG1914	+
contig 30	1931	1917	0,7	142	511	47	400	60	68	119	6	30	1137	1E-153	NANOZOOG1927	+
contig 30	1960	2182	1,8	24	830	1	736	80	89	93	6	71	2959	0E+00	NANOZOOG1956	+
contig 31	2182	1960	1,8	1	736	24	830	80	56	89	6	71	3017	0E+00	NANOZOOG2177	+
contig 30	1960	5744	1,8	24	830	1	776	83	93	103	4	31	3158	0E+00	NANOZOOG1956	+
contig 66	5744	1960	1,8	24	830	1	776	83	93	103	4	31	3158	0E+00	NANOZOOG5732	+
contig 31	2140	2163	1,8	1	856	1	837	91	97	52	2	25	4077	0E+00	NANOZOOG2135	+
contig 31	2163	2140	1,8	1	837	1	856	91	100	52	2	25	4077	0E+00	NANOZOOG2158	+
contig 31	2182	5744	1,8	1	1301	1	1340	85	98	130	7	75	5228	0E+00	NANOZOOG2177	+
contig 66	5744	2182	1,8	1	1340	1	1301	85	96	129	7	75	5233	0E+00	NANOZOOG5732	+
contig 38	2814	2815	1,1	2	354	1	366	96	99	0	1	13	1752	0E+00	NANOZOOG2808	-
contig 38	2815	2814	1,1	1	366	2	354	96	96	0	1	13	1752	0E+00	NANOZOOG2809	+
contig 38	3048	3049	1,1	460	804	1	345	100	41	0	0	0	1848	0E+00	NANOZOOG3042	-
contig 38	3049	3048	1,1	1	345	460	804	100	100	0	0	0	1848	0E+00	NANOZOOG3043	+
contig 38	3071	3073	0,4	4	156	15	161	95	11	2	1	6	624	3E-74	NANOZOOG3065	-
contig 38	3073	3071	0,4	12	161	1	156	95	68	2	1	6	698	3E-85	NANOZOOG3067	+
contig 39	3191	3192	1,3	98	659	1	534	93	76	12	3	28	2647	0E+00	NANOZOOG3184	-
contig 39	3192	3191	1,3	1	534	98	659	93	100	12	3	28	2647	0E+00	NANOZOOG3185	+
contig 39	3207	3228	0,2	1	258	82	334	38	96	146	8	17	415	2E-49	NANOZOOG3200	+
contig 39	3228	3207	0,2	82	334	1	258	38	74	149	7	15	371	8E-43	NANOZOOG3221	+
contig 39	3262	3263	1,1	24	450	1	421	79	92	73	3	16	1672	0E+00	NANOZOOG3255	-
contig 39	3263	3262	1,1	1	421	24	450	79	99	73	3	16	1662	0E+00	NANOZOOG3256	-
contig 10	38	24	1,4	1	578	1	602	74	75	131	3	24	2256	0E+00	NANOZOOG39	+
contig 10	24	38	1,4	1	602	1	578	74	92	131	3	24	2222	0E+00	NANOZOOG25	-
contig 45	3996	4172	1,2	1	394	106	499	93	100	27	0	0	1889	0E+00	NANOZOOG3989	-
contig 46	4172	3996	1,2	106	499	1	394	93	41	27	0	0	1889	0E+00	NANOZOOG4164	+
contig 45	3997	4172	0,1	88	116	1	29	97	25	1	0	0	147	1E-12	NANOZOOG3990	-
contig 46	4172	3997	0,1	1	29	88	116	97	3	1	0	0	147	9E-12	NANOZOOG4164	+
contig 45	3999	4000	0,3	310	403	1	94	100	8	0	0	0	496	4E-59	NANOZOOG3992	+
contig 45	4000	3999	0,3	1	94	310	403	100	100	0	0	0	496	4E-60	NANOZOOG3993	-
contig 45	3999	645	0,4	590	1109	743	1251	36	45	318	10	15	784	3E-87	NANOZOOG3992	+
				147	536	218	607	31	34	254	11	26	383	1E-37		
contig 11	645	3999	0,4	743	1251	590	1109	37	40	311	11	23	785	2E-87	NANOZOOG645	+
				218	607	147	536	30	30	255	10	26	380	3E-37		
contig 45	4016	4020	1,1	1	381	1	429	84	98	21	3	48	1783	0E+00	NANOZOOG4008	+
contig 45	4020	4016	1,1	1	437	1	389	84	89	22	3	48	1815	0E+00	NANOZOOG4012	-
contig 45	4044	4049	1,5	1	562	1	540	96	96	1	1	22	2725	0E+00	NANOZOOG4036	+
contig 45	4049	4044	1,5	1	540	1	562	96	93	1	1	22	2725	0E+00	NANOZOOG4041	-
contig 5	4319	4337	0,9	85	390	23	320	94	76	9	1	8	1470	0E+00	NANOZOOG4311	-
contig 5	4337	4319	0,9	23	320	85	390	94	93	9	1	8	1470	0E+00	NANOZOOG4329	+

contig 5	4320	4333	1,8	1324	2016	1540	2279	89	33	3	3	83	3530	0E+00	NANOZOOG4312	-
				693	1206	865	1314	82	22	26	4	66	1994	0E+00		
				1	296	123	418	100	15	1	0	0	1641	0E+00		
				297	526	562	791	99	11	3	0	0	1172	1E-130		
contig 5	4333	4320	1,8	1540	2279	1324	2016	89	30	3	3	83	3522	0E+00	NANOZOOG4325	+
				862	1319	690	1211	81	20	33	3	64	2004	0E+00		
				123	418	1	296	100	13	1	0	0	1638	0E+00		
				562	791	297	526	99	10	3	0	0	1169	2E-130		
contig 5	4350	4351	0,5	1	161	1	161	100	74	0	0	0	832	2E-113	NANOZOOG4342	-
contig 5	4351	4350	0,5	1	161	1	161	100	55	0	0	0	833	1E-113	NANOZOOG4343	+
contig 5	4513	4514	0,3	52	162	1	111	100	59	0	0	0	556	9E-75	NANOZOOG4503	-
contig 5	4514	4513	0,3	1	111	52	162	100	100	0	0	0	556	5E-75	NANOZOOG4504	-
contig 5	4606	4608	1,6	56	519	67	530	99	89	6	0	0	2485	0E+00	NANOZOOG4596	+
contig 5	4608	4606	1,6	67	530	56	519	99	88	6	0	0	2485	0E+00	NANOZOOG4597	-
contig 5	4665	5915	0,5	1	364	21	377	50	95	164	10	23	815	2E-107	NANOZOOG4654	-
contig 7	5915	4665	0,5	21	377	1	364	50	93	162	11	23	832	7E-110	NANOZOOG5903	+
contig 5	4799	4806	0,6	5	359	1	357	69	93	72	7	46	1096	3E-150	NANOZOOG4788	+
contig 5	4806	4799	0,6	1	357	5	359	69	93	73	6	42	1110	2E-152	NANOZOOG4795	-
contig 5	4820	4821	1,8	1	577	93	669	100	65	1	0	0	3107	0E+00	NANOZOOG4809	-
				452	735	1	305	25	31	202	5	31	226	2E-19		
contig 5	4821	4820	1,8	93	669	1	577	100	86	1	0	0	3107	0E+00	NANOZOOG4810	+
				1	305	452	735	25	42	202	5	31	226	2E-19		
contig 5	4904	6916	0,5	1	300	15	318	57	94	114	7	22	841	6E-113	NANOZOOG4893	-
scaffold 49	6916	4904	0,5	15	318	1	300	57	88	114	7	22	841	6E-113	NANOZOOG6904	+
contig 5	4985	6431	1,8	4	550	127	633	66	49	140	4	50	1774	0E+00	NANOZOOG4974	-
				599	1033	722	1218	64	42	118	5	62	1600	0E+00		
scaffold 40	6431	4985	1,8	127	633	4	550	66	33	140	4	50	1821	0E+00	NANOZOOG6419	-
				722	1218	599	1033	64	28	118	5	62	1601	0E+00		
contig 62	5301	5315	0,2	2	209	7	165	41	60	73	4	49	363	1E-43	NANOZOOG5290	-
contig 62	5315	5301	0,2	7	165	2	209	41	96	73	4	49	362	1E-43	NANOZOOG5304	-
contig 62	5361	5364	1,0	1	359	30	375	87	92	1	6	47	1671	0E+00	NANOZOOG5350	-
contig 62	5364	5361	1,0	30	375	1	359	87	88	1	6	47	1671	0E+00	NANOZOOG5353	+
contig 65	5478	5479	0,6	232	536	1	305	100	57	0	0	0	1542	0E+00	NANOZOOG5467	+
contig 65	5479	5478	0,6	1	305	232	536	100	100	0	0	0	1542	0E+00	NANOZOOG5468	-
contig 65	5489	5490	1,8	50	611	1	552	98	66	1	1	10	2919	0E+00	NANOZOOG5478	-
contig 65	5490	5489	1,8	1	552	50	611	98	100	1	1	10	2919	0E+00	NANOZOOG5479	+
contig 7	5795	5796	1,8	100	917	1	818	100	60	0	0	0	4320	0E+00	NANOZOOG5783	+
contig 7	5796	5795	1,8	1	818	100	917	100	100	0	0	0	4320	0E+00	NANOZOOG5784	-
scaffold 40	6488	6489	1,8	532	1212	1	615	87	38	18	5	72	3034	0E+00	NANOZOOG6476	+
scaffold 40	6489	6488	1,8	1	615	532	1212	87	100	18	5	72	3034	0E+00	NANOZOOG6477	-
scaffold 40	6541	6542	0,8	5	260	1	256	100	98	1	0	0	1340	0E+00	NANOZOOG6529	+
scaffold 40	6542	6541	0,8	1	256	5	260	100	99	1	0	0	1340	0E+00	NANOZOOG6530	-
contig 11	662	663	0,3	2	137	1	178	75	99	2	1	42	621	6E-84	NANOZOOG662	-

contig 11	663	662	0,3	1	178	2	137	75	60	3	2	42	628	9E-85	NANOZOOG663	+
scaffold 40	6688	6689	0,5	117	340	1	224	100	64	0	0	0	1147	2E-160	NANOZOOG6676	+
scaffold 40	6689	6688	0,5	1	224	117	340	100	100	0	0	0	1147	1E-160	NANOZOOG6677	-
scaffold 49	6849	6850	0,4	237	413	3	179	100	39	0	0	0	887	6E-120	NANOZOOG6837	+
scaffold 49	6850	6849	0,4	3	179	237	413	100	99	0	0	0	887	2E-120	NANOZOOG6838	-
scaffold 49	6968	6969	1,0	1	302	1	302	100	91	1	0	0	1611	0E+00	NANOZOOG6956	+
scaffold 49	6969	6968	1,0	1	302	1	302	100	100	1	0	0	1611	0E+00	NANOZOOG6957	-
scaffold 6	7285	7300	0,3	52	162	1	111	100	69	0	0	0	554	7E-75	NANOZOOG7271	-
scaffold 6	7300	7285	0,3	1	111	52	162	100	100	0	0	0	554	4E-75	NANOZOOG7286	-
scaffold 6	7286	7301	0,3	31	144	1	114	100	73	0	0	0	590	3E-80	NANOZOOG7272	+
scaffold 6	7301	7286	0,3	1	114	31	144	100	90	0	0	0	590	2E-80	NANOZOOG7287	-
scaffold 6	7312	7313	1,8	461	1837	1	1371	100	74	0	2	6	7255	0E+00	NANOZOOG7298	-
scaffold 6	7313	7312	1,8	1	1371	461	1837	100	100	0	2	6	7255	0E+00	NANOZOOG7299	+

Supplementary Table S11. Alignment statistics of the duplicated genes identified in the *Rhodotorula babjevae* DBVPG 8058 genome by OrthoVenn2

Contig/ scaffold	Query	Subject	inflation	Query start	Query end	Subject start	Subject end	% identity	% coverage	Mismatches	Gaps	Gap Bases	Score	E-Value	Gene ID	Strand
contig 1	7	8	0,7	1	215	65	279	100	73	0	0	0	1152	3E-161	NANOZOOG8	-
contig 1	8	7	0,7	65	279	1	215	100	77	0	0	0	1152	3E-161	NANOZOOG9	+
contig 33	450	490	0,1	1	87	10	79	45	47	31	1	17	189	3E-19	NANOZOOG450	-
contig 33	490	450	0,1	10	79	1	87	45	47	31	1	17	185	9E-19	NANOZOOG490	-
contig 38	699	700	0,6	4	341	1	382	53	98	129	4	54	982	4E-133	NANOZOOG699	-
contig 38	700	699	0,6	1	382	4	341	53	87	129	4	54	982	5E-133	NANOZOOG700	-
contig 38	881	918	0,3	1	131	1	131	99	100	1	0	0	657	8E-91	NANOZOOG881	+
contig 38	918	881	0,3	1	131	1	131	99	96	1	0	0	657	8E-91	NANOZOOG918	+
contig 40	1190	1191	0,8	117	447	1	333	99	74	0	1	2	1702	0E+00	NANOZOOG1189	+
contig 40	1191	1190	0,8	1	333	117	447	99	99	0	1	2	1702	0E+00	NANOZOOG1190	-

contig 42	1575	1576	1,0	22	597	2	623	54	95	238	5	52	1710	0E+00	NANOZOOG1573	+
contig 42	1576	1575	1,0	13	623	33	597	54	90	232	5	52	1694	0E+00	NANOZOOG1574	+
contig 42	1638	1639	0,2	3	90	1	88	98	63	2	0	0	443	1E-58	NANOZOOG1636	+
contig 42	1639	1638	0,2	1	88	3	90	98	100	2	0	0	443	6E-59	NANOZOOG1637	-
contig 42	1914	1926	1,1	1	581	1	621	66	78	167	6	42	1547	0E+00	NANOZOOG1911	-
				632	733	768	869	81	14	19	0	0	420	4E-43		
contig 42	1926	1914	1,1	1	621	1	581	66	66	171	5	42	1545	0E+00	NANOZOOG1923	+
				768	882	632	742	79	13	20	1	4	437	3E-45		
contig 42	2011	2024	0,2	1	280	1	278	40	98	161	4	10	352	2E-40	NANOZOOG2008	+
contig 42	2024	2011	0,2	1	278	1	280	35	94	156	5	36	354	8E-41	NANOZOOG2021	-
contig 45	2239	4377	0,3	1	236	1	227	49	96	112	3	9	489	1E-60	NANOZOOG2235	+
contig 65	4377	2239	0,3	1	227	1	236	49	64	112	3	9	486	4E-60	NANOZOOG4367	+
contig 47	2581	2590	1,6	1	800	1	754	67	93	206	9	64	2524	0E+00	NANOZOOG2573	-
contig 47	2590	2581	1,6	1	541	1	541	79	71	110	2	2	2102	0E+00	NANOZOOG2582	-
				609	754	631	800	57	19	49	1	24	507	2E-54		
contig 47	2601	2627	0,8	1	414	1	343	68	82	59	6	75	1356	0E+00	NANOZOOG2593	+
contig 47	2627	2601	0,8	1	343	1	414	68	99	60	6	75	1324	0E+00	NANOZOOG2619	+
contig 47	2722	2726	1,1	17	419	1	395	88	90	25	4	24	1764	0E+00	NANOZOOG2714	-
contig 47	2726	2722	1,1	1	395	17	419	87	98	30	4	24	1687	0E+00	NANOZOOG2718	+
contig 48	2976	2977	0,9	664	1001	1	338	100	29	0	0	0	1753	0E+00	NANOZOOG2967	-
contig 48	2977	2976	0,9	1	338	664	1001	100	100	0	0	0	1753	0E+00	NANOZOOG2968	+
contig 48	3002	3004	0,3	291	456	99	237	64	30	32	1	27	573	1E-71	NANOZOOG2993	+
contig 48	3004	3002	0,3	57	237	238	456	56	73	58	2	38	595	3E-75	NANOZOOG2995	+
contig 51	3445	3446	1,8	749	1312	91	654	100	33	1	0	0	2994	0E+00	NANOZOOG3436	-
				560	650	1	91	100	5	0	0	0	487	2E-51		
contig 51	3446	3445	1,8	91	654	749	1312	100	86	1	0	0	2994	0E+00	NANOZOOG3437	+
				1	91	560	650	100	14	0	0	0	487	9E-52		
contig 51	3500	4822	1,8	711	1428	683	1451	54	48	261	11	109	2049	0E+00	NANOZOOG3491	+
				31	470	24	403	57	26	125	7	68	1157	9E-135		
				572	701	404	575	45	9	53	1	42	359	2E-34		
contig 69	4822	3500	1,8	683	1239	711	1222	67	35	140	2	45	1964	0E+00	NANOZOOG4811	+
				24	403	31	470	57	26	125	7	68	1154	3E-134		
				404	575	572	701	45	9	53	1	42	310	1E-28		
contig 53	3517	7028	0,7	1	224	29	252	98	100	5	0	0	1118	1E-157	NANOZOOG3508	-
contig 86	7028	3517	0,7	29	252	1	224	98	89	5	0	0	1118	1E-157	NANOZOOG6994	-
contig 69	4979	4980	1,8	1	620	51	670	100	93	1	0	0	3319	0E+00	NANOZOOG4967	+
				505	669	6	175	28	24	114	3	9	175	2E-13		
contig 69	4980	4979	1,8	51	670	1	620	100	93	1	0	0	3319	0E+00	NANOZOOG4968	-
				6	175	505	669	28	24	114	3	9	175	2E-13		
contig 69	5060	5062	1,8	204	999	2	956	76	80	69	9	159	3159	0E+00	NANOZOOG5048	+
contig 69	5062	5060	1,8	2	956	204	999	76	83	69	9	159	3148	0E+00	NANOZOOG5049	-
contig 70	5308	5318	0,3	1	122	1	122	100	76	0	0	0	620	8E-85	NANOZOOG5294	-
contig 70	5318	5308	0,3	1	122	1	122	100	100	0	0	0	620	6E-85	NANOZOOG5304	+

contig 70	5308	6580	0,3	5	159	1	156	84	96	20	2	5	615	1E-83	NANOZOOG5294	-
contig 82	6580	5308	0,3	1	156	5	159	84	97	21	2	5	597	7E-81	NANOZOOG6562	+
contig 70	5329	5332	1,8	156	1230	1	1061	99	50	0	1	14	5626	0E+00	NANOZOOG5315	-
contig 70	5332	5329	1,8	1	1061	156	1230	99	100	0	1	14	5626	0E+00	NANOZOOG5318	+
contig 73	5763	5786	1,8	77	786	1	709	89	84	31	3	47	3375	0E+00	NANOZOOG5747	+
contig 73	5786	5763	1,8	1	709	77	786	89	97	31	3	47	3376	0E+00	NANOZOOG5770	+
contig 74	5904	5905	0,5	270	534	1	265	100	49	0	0	0	1317	0E+00	NANOZOOG5888	+
contig 74	5905	5904	0,5	1	265	270	534	100	100	0	0	0	1317	0E+00	NANOZOOG5889	-
contig 74	5915	5916	1,8	50	609	1	549	98	59	1	1	11	2905	0E+00	NANOZOOG5899	-
contig 74	5916	5915	1,8	1	549	50	609	98	100	1	1	11	2905	0E+00	NANOZOOG5900	+
contig 77	6501	6510	1,8	1	750	1	703	91	94	18	2	47	3469	0E+00	NANOZOOG6483	-
contig 77	6510	6501	1,8	1	703	1	750	91	100	18	2	47	3469	0E+00	NANOZOOG6492	+
contig 84	6685	6687	0,7	2	464	3	482	53	98	200	4	29	1155	1E-155	NANOZOOG6667	-
contig 84	6687	6685	0,7	3	482	2	464	53	94	194	5	37	1214	9E-165	NANOZOOG6669	-
scaffold 52	7488	7489	1,2	80	443	1	368	99	81	0	2	4	1911	0E+00	NANOZOOG7454	+
scaffold 52	7489	7488	1,2	1	368	80	443	99	99	0	2	4	1911	0E+00	NANOZOOG7455	-
scaffold 52	7497	7498	1,4	1	501	10	515	99	100	0	1	5	2509	0E+00	NANOZOOG7463	-
scaffold 52	7498	7497	1,4	10	515	1	501	99	97	0	1	5	2509	0E+00	NANOZOOG7464	+

Supplementary Table S12. Alignment statistics of the duplicated genes identified in the *Rhodotorula babjevae* CBS 7808 genome by BLASTp

Contig/ scaffold	Query	Subject	Query start	Query stop	Subject start	Subject stop	% Identity	% coverage	Mismatches	Gaps	Gap Bases	Score	E-Value	Gene ID	Strad
contig 10	24	38	1	602	1	578	74	92	131	3	24	2222	0E+00	NANOZOOG25	-
contig 10	38	24	1	578	1	602	74	75	131	3	24	2256	0E+00	NANOZOOG39	+
contig 10	84	701	12	359	1	401	68	94	66	7	63	1338	0E+00	NANOZOOG85	-
contig 11	701	84	1	401	12	359	68	84	66	7	63	1338	0E+00	NANOZOOG701	-
contig 10	122	123	8	190	1	211	84	91	5	1	28	873	2E-121	NANOZOOG123	+
contig 10	123	122	1	211	8	190	87	87	0	1	28	905	3E-126	NANOZOOG124	-
contig 10	326	327	1	330	1	359	73	94	67	4	29	1258	3E-174	NANOZOOG326	-
contig 10	327	326	1	359	1	330	72	79	70	4	29	1260	2E-174	NANOZOOG327	-
contig 12	801	812	1	280	1	304	89	82	8	1	24	1379	0E+00	NANOZOOG801	+
contig 12	812	801	1	304	1	280	89	91	8	1	24	1379	0E+00	NANOZOOG812	-
contig 12	830	831	13	178	1	166	100	93	0	0	0	894	1E-125	NANOZOOG830	+
contig 12	831	830	1	166	13	178	100	97	0	0	0	894	1E-125	NANOZOOG831	-
contig 30	1409	5744	1	1354	3	1340	90	99	114	4	20	5728	0E+00	NANOZOOG1409	-
contig 66	5744	1409	3	1340	1	1354	90	100	112	4	20	5706	0E+00	NANOZOOG5732	+
contig 66	5744	2182	1	1340	1	1301	85	96	129	7	75	5233	0E+00	NANOZOOG5732	+
contig 31	2182	5744	1	1301	1	1340	85	98	130	7	75	5228	0E+00	NANOZOOG2177	+
contig 30	1409	2182	1	1354	3	1301	87	96	112	5	59	5497	0E+00	NANOZOOG1409	-
contig 31	2182	1409	3	1301	1	1354	87	99	111	5	59	5492	0E+00	NANOZOOG2177	+
contig 30	1471	1488	73	364	1	256	87	70	3	2	36	1222	5E-171	NANOZOOG1470	-
contig 30	1488	1471	1	256	73	364	87	100	3	2	36	1222	4E-171	NANOZOOG1487	+
contig 30	1500	1501	15	276	30	296	76	94	55	4	9	1038	9E-144	NANOZOOG1499	-
contig 30	1501	1500	30	296	15	276	76	86	55	4	9	1037	1E-143	NANOZOOG1500	-
contig 30	1515	1516	1	189	31	219	100	99	0	0	0	983	3E-138	NANOZOOG1514	+
contig 30	1516	1515	31	219	1	189	100	86	0	0	0	983	3E-138	NANOZOOG1515	-
contig 30	1851	1892	42	284	1	209	84	70	1	4	38	992	6E-138	NANOZOOG1848	-
contig 30	1892	1851	1	209	42	284	84	99	1	4	38	992	4E-138	NANOZOOG1889	-
contig 31	2140	2163	1	856	1	837	91	97	52	2	25	4077	0E+00	NANOZOOG2135	+
contig 31	2163	2140	1	837	1	856	91	100	52	2	25	4077	0E+00	NANOZOOG2158	+
contig 36	2615	2616	16	325	1	266	86	75	0	1	44	959	5E-131	NANOZOOG2609	+
contig 36	2616	2615	1	266	16	325	86	100	0	1	44	964	6E-132	NANOZOOG2610	-
contig 37	2717	2727	1	574	1	627	79	100	80	2	53	2379	0E+00	NANOZOOG2711	-
contig 37	2727	2717	1	627	1	574	79	92	77	2	53	2408	0E+00	NANOZOOG2721	+
contig 38	2814	2815	2	354	1	366	96	99	0	1	13	1752	0E+00	NANOZOOG2808	-
contig 38	2815	2814	1	366	2	354	96	96	0	1	13	1752	0E+00	NANOZOOG2809	+
contig 38	2854	2877	1	481	1	476	85	97	67	2	7	1475	0E+00	NANOZOOG2848	-
contig 38	2877	2854	1	469	1	474	85	78	66	2	7	1502	0E+00	NANOZOOG2871	+
contig 38	3041	3043	153	610	3	460	100	72	1	0	0	2369	0E+00	NANOZOOG3035	+
contig 38	3043	3041	3	460	153	610	100	100	1	0	0	2369	0E+00	NANOZOOG3037	-
contig 39	3191	3192	98	659	1	534	93	76	12	3	28	2647	0E+00	NANOZOOG3184	-
contig 39	3192	3191	1	534	98	659	93	100	12	3	28	2647	0E+00	NANOZOOG3185	+
contig 39	3262	3263	24	450	1	421	79	92	73	3	16	1672	0E+00	NANOZOOG3255	-
contig 39	3263	3262	1	421	24	450	79	99	73	3	16	1662	0E+00	NANOZOOG3256	-
contig 45	4016	4020	1	381	1	429	84	98	21	3	48	1783	0E+00	NANOZOOG4008	+
contig 45	4020	4016	1	437	1	389	84	89	22	3	48	1815	0E+00	NANOZOOG4012	-
contig 45	4044	4049	1	562	1	540	96	96	1	1	22	2725	0E+00	NANOZOOG4036	+
contig 45	4049	4044	1	540	1	562	96	93	1	1	22	2725	0E+00	NANOZOOG4041	-
contig 5	4319	4337	85	390	23	320	94	76	9	1	8	1470	0E+00	NANOZOOG4311	-
contig 5	4337	4319	23	320	85	390	94	93	9	1	8	1470	0E+00	NANOZOOG4329	+

contig 5	4341	4342	16	520	1	495	98	95	0	1	10	2477	0E+00	NANOZOOG4333	+
contig 5	4342	4341	1	495	16	520	98	100	0	1	10	2477	0E+00	NANOZOOG4334	-
contig 62	5361	5364	1	359	30	375	87	92	1	6	47	1671	0E+00	NANOZOOG5350	-
contig 62	5364	5361	30	375	1	359	87	88	1	6	47	1671	0E+00	NANOZOOG5353	+
contig 7	5765	5781	2	401	85	501	78	89	72	3	19	1543	0E+00	NANOZOOG5753	+
contig 7	5781	5765	84	501	1	401	78	80	72	3	19	1590	0E+00	NANOZOOG5769	-
contig 7	5929	5930	1	444	147	590	100	100	0	0	0	2286	0E+00	NANOZOOG5917	+
contig 7	5930	5929	147	590	1	444	100	75	0	0	0	2286	0E+00	NANOZOOG5918	-
scaffold 40	6541	6542	5	260	1	256	100	98	1	0	0	1340	0E+00	NANOZOOG6529	+
scaffold 40	6542	6541	1	256	5	260	100	99	1	0	0	1340	0E+00	NANOZOOG6530	-
scaffold 49	6968	6969	1	302	1	302	100	91	1	0	0	1611	0E+00	NANOZOOG6956	+
scaffold 49	6969	6968	1	302	1	302	100	100	1	0	0	1611	0E+00	NANOZOOG6957	-
scaffold 6	7144	7145	9	163	1	153	95	91	5	1	2	767	1E-106	NANOZOOG7132	+
scaffold 6	7145	7144	1	153	9	163	95	100	5	1	2	767	1E-106	NANOZOOG7133	+
scaffold 6	7266	7271	1	353	76	407	86	93	24	2	27	1381	0E+00	NANOZOOG7252	+
scaffold 6	7271	7266	76	407	1	353	86	81	24	2	27	1405	0E+00	NANOZOOG7257	-
scaffold 6	7312	7313 2	461	1837	1	1371	100	74	0	2	6	7255	0E+00	NANOZOOG7298	-
scaffold 6	7313	7312	1	257	196	452	100	97	0	0	0	1350	2E-174	NANOZOOG7299	+
contig 5	4606	4608	56	519	67	530	99	89	6	0	0	2485	0E+00	NANOZOOG4596	+
contig 5	4608	4606	67	530	56	519	99	88	6	0	0	2485	0E+00	NANOZOOG4597	-
contig 28	1276	1280	1	590	1	595	80	96	71	7	55	2390	0E+00	NANOZOOG1276	-
contig 28	1280	1276	1	595	1	590	80	95	71	8	51	2317	0E+00	NANOZOOG1280	+
scaffold 6	7498	7497	1	399	1	355	89	78	0	1	44	1870	0E+00	NANOZOOG7483	+
scaffold 6	7497	7498	1	355	1	399	89	100	0	1	44	1870	0E+00	NANOZOOG7482	-

Supplementary Table S13. Alignment statistics of the duplicated genes identified in the *Rhodotorula babjevae* DBVPG 8058 genome by BLASTp

Contig/ scaffold	Query	Subject	Query start	Query stop	Subject start	Subject stop	% identity	% coverage	Mismatches	Gaps	Gap Bases	Score	E-Value	Gene ID	Sense
contig 1	7	8	1	215	65	279	100	73	0	0	0	1152	3E-161	NANOZOOG8	-
contig 1	8	7	65	279	1	215	100	77	0	0	0	1152	3E-161	NANOZOOG9	+
contig 40	1190	1191	117	447	1	333	99	74	0	1	2	1702	0E+00	NANOZOOG1189	+
contig 40	1191	1190	1	333	117	447	99	99	0	1	2	1702	0E+00	NANOZOOG1190	-
contig 42	1785	1823	21	253	1	254	86	84	6	2	29	901	2E-123	NANOZOOG1782	+
contig 42	1823	1785	1	254	21	253	86	85	6	2	29	966	2E-133	NANOZOOG1820	+
contig 45	2269	2270	1	341	74	414	98	80	8	0	0	1769	0E+00	NANOZOOG2265	+
contig 45	2270	2269	74	414	1	341	98	82	8	0	0	1769	0E+00	NANOZOOG2266	-
contig 47	2722	2726	17	419	1	395	88	90	25	4	24	1764	0E+00	NANOZOOG2714	-
contig 47	2726	2722	1	395	17	419	87	98	30	4	24	1687	0E+00	NANOZOOG2718	+
contig 48	2853	2854	137	580	1	444	100	77	0	0	0	2285	0E+00	NANOZOOG2845	+
contig 48	2854	2853	1	444	137	580	100	100	0	0	0	2285	0E+00	NANOZOOG2846	-
contig 51	3245	3246	1	187	36	222	100	94	0	0	0	932	3E-130	NANOZOOG3236	-
contig 51	3246	3245	36	222	1	187	100	84	0	0	0	932	4E-130	NANOZOOG3237	+
contig 51	3354	3355	98	459	1	359	99	76	1	2	3	1900	0E+00	NANOZOOG3345	+
contig 51	3355	3354	1	359	98	459	99	100	1	2	3	1900	0E+00	NANOZOOG3346	-
contig 51	3393	3394	1	233	37	269	100	93	1	0	0	1221	1E-172	NANOZOOG3384	+
contig 51	3394	3393	37	269	1	233	100	87	1	0	0	1221	1E-172	NANOZOOG3385	-
contig 53	3517	7028	1	224	29	252	98	100	5	0	0	1118	1E-157	NANOZOOG3508	-
contig 86	7028	3517	29	252	1	224	98	89	5	0	0	1118	1E-157	NANOZOOG6994	-
contig 53	3695	3696	1	173	37	209	100	97	0	0	0	910	2E-127	NANOZOOG3686	+
contig 53	3696	3695	37	209	1	173	100	83	0	0	0	910	2E-127	NANOZOOG3687	-
contig 54	3739	3743	1	439	1	364	75	82	34	4	75	1598	0E+00	NANOZOOG3730	+
contig 54	3743	3739	1	361	1	436	75	99	36	4	75	1486	0E+00	NANOZOOG3734	-
contig 69	4979	4980	1	620	51	670	100	93	1	0	0	3319	0E+00	NANOZOOG4967	+
contig 69	4980	4979	51	670	1	620	100	93	1	0	0	3319	0E+00	NANOZOOG4968	-
contig 69	5060	5062	204	999	2	956	76	80	69	9	159	3159	0E+00	NANOZOOG5048	+
contig 69	5062	5060	2	956	204	999	76	83	69	9	159	3148	0E+00	NANOZOOG5049	-
contig 70	5308	6580	5	159	1	156	84	96	20	2	5	615	1E-83	NANOZOOG5294	-
contig 82	6580	5308	1	156	5	159	84	97	21	2	5	597	7E-81	NANOZOOG6562	+
contig 71	5441	5451	1	475	1	475	87	83	62	0	0	2050	0E+00	NANOZOOG5427	-
contig 71	5451	5441	1	475	1	475	87	100	62	0	0	2113	0E+00	NANOZOOG5437	+
contig 73	5763	5786	77	786	1	709	89	84	31	3	47	3375	0E+00	NANOZOOG5747	+
contig 73	5786	5763	1	709	77	786	89	97	31	3	47	3376	0E+00	NANOZOOG5770	+
contig 75	6248	6249	7	379	1	363	97	93	0	1	10	1907	0E+00	NANOZOOG6230	-
contig 75	6249	6248	1	363	7	379	97	100	0	1	10	1907	0E+00	NANOZOOG6231	+
contig 77	6501	6510	1	750	1	703	91	94	18	2	47	3469	0E+00	NANOZOOG6483	-
contig 77	6510	6501	1	703	1	750	91	100	18	2	47	3469	0E+00	NANOZOOG6492	+
scaffold 52	7488	7489	80	443	1	368	99	81	0	2	4	1911	0E+00	NANOZOOG7454	+
scaffold 52	7489	7488	1	368	80	443	99	99	0	2	4	1911	0E+00	NANOZOOG7455	-
scaffold 52	7497	7498	1	501	10	515	99	100	0	1	5	2509	0E+00	NANOZOOG7463	-
scaffold 52	7498	7497	10	515	1	501	99	97	0	1	5	2509	0E+00	NANOZOOG7464	+

Supplementary Table S14. Duplicated genes in *Rhodotorula babjevae* identified by BLASTp and OrthoVenn2 with a minimum coverage of 70%

Genetic structure	CBS 7808	Functional prediction	DBVPG 8058	Functional prediction
**Putative chromosome 1**	4319/4337	Protein bcp1	4979/4980	Carbamoyl-phosphate synthase arginine-specific large chain
	4341/4342	Ser/Arg repetitive matrix protein 2	5060/5062	Ser/Arg repetitive matrix protein 2 & Pantothenate transporter liz1
	4606/4608	Phenylalanine--tRNA ligase alpha subunit & Probable feruloyl esterase B-2	7488/7489	EF-1-alpha
	4665	Uncharacterized protein C17G8.02	7497/7498	Glycoprotein gp2
	4799/4806	Immediate-early protein 2 & Putative uncharacterized protein ENSP00000383309		
	4904	A-agglutinin anchorage subunit		
**Putative chromosome 2**	2814/2815	Ser/Arg repetitive matrix protein 1	699/700	Quinone-oxidoreductase homolog, chloroplastic & MUC-5AC
	2854/2877	Putative uncharacterized protein ENSP00000383309 & Ser/Arg repetitive matrix protein 3	881/918	Histone H2A
	3041/3043	Heat shock 70 kDa protein & Heat shock protein SSC1, mitochondrial		
	5361/5364	Protein arginine N-methyltransferase 1		
**Putative chromosome 3**	1409	Ser/Arg repetitive matrix protein 1	1575/1776*	Cytochrome P450 monooxygenase ALT8
	1471/1488	Ser/Arg repetitive matrix protein 2 & Protein YIP5	1785/1823*	MUC-5AC & Pre-mRNA-splicing factor CWC22
	1500/1501	Putative protein TPRXL	2011/2024*	Ser/Arg repetitive matrix protein 2 & AtPERK9
	1515/1516	Ser/Arg repetitive matrix protein 2		
	1749/1763	Protein SON & Pre-mRNA-splicing factor CWC21		
	1851/1892	RNA-binding protein with serine-rich domain 1 & ERF3		
	1960	Pneumococcal serine-rich repeat protein		
**Putative chromosome 4**			6248/6249	IE2
**Putative chromosome 5**	5765/5781	Ser/Arg repetitive matrix protein 2 & Ser/Arg repetitive matrix protein 1	2853/2854	Heat shock protein 60, mitochondrial
	5915	Uncharacterized protein C17G8.02	3517	Uncharacterized serine-rich protein C215.13
	5929/5930	Heat shock protein 60, mitochondrial	3695/3696	40S ribosomal protein S1
**Putative chromosome 6**	701	Alpha-ketoglutarate-dependent sulfonate dioxygenase	1575/1776*	Cytochrome P450 monooxygenase ALT8
			1785/1823*	MUC-5AC & Pre-mRNA-splicing factor CWC22
			2011/2024*	Ser/Arg repetitive matrix protein 2 & AtPERK9
			6681/6687	Endochitinase 2 & Glycoprotein gp2
	7144/7145	Tryptophan synthase	5308/5318	Histone H3.2

**Putative chromosome 7**	7266/7271	Mannose-6-phosphate isomerase		
	7286/7301	Histone H3.2		
	7312/7313	Acetyl-CoA carboxylase		
	7498/7497	Sulfite reductase [NADPH] hemoprotein beta-component & Sulfite reductase [NADPH] subunit beta		
**Putative chromosome 8**	6916	Chitin deacetylase 1	1190/1191	AtLRX3
	6968/6969	60S ribosomal protein L3		
**Putative chromosome 9**	24/38	Ser/Arg repetitive matrix protein 2 & Vitellogenin-1	2581/2590	Ser/Arg repetitive matrix protein 2
	84	Alpha-ketoglutarate-dependent dioxygenase cnsM	2601/2627	MUC-5AC
	122/123	MUC-5AC & Ser/Arg-rich splicing factor SR45	2722/2726	Ser/Arg repetitive matrix protein 1
	326/327	Ser/Arg repetitive matrix protein 2 & Ser/Arg repetitive matrix protein 1	3739/3743*	Trimethylguanosine synthase
**Putative chromosome 10**	2615/2616	Ser/Arg repetitive matrix protein 1		
**Putative chromosome 11**	2140/2163	MUC-5AC & AtOPT4	5763/5786	AtOPT4
	2182	Ser/Arg repetitive matrix protein 1	6580	Histone H3
**Putative chromosome 12**	6541/6542	Fumarate hydratase, mitochondrial	3245/3246	Splicing factor YJU2 & Protamine
			3354/3355	Actin
			3393/3394	Fumarate hydratase, mitochondrial
**Putative chromosome 13**	801/812	Ser/Arg repetitive matrix protein 2	5441/5451	Ser/Arg repetitive matrix protein 2
	830/831	Phosphoenolpyruvate carboxykinase (ATP)		
	2717/2727	Ser/Arg repetitive matrix protein 2 & Putative protein TPRXL		
**Putative chromosome 14**	4016/4020	Trimethylguanosine synthase	6501/6510	Putative GPI-anchored protein pfl2
	4044/4049	Branched chain 2-oxo-acid dehydrogenase complex component E2	3739/3743*	Trimethylguanosine synthase
**Putative chromosome 17**	3191/3192	Ser/Arg repetitive matrix protein 1	07/08	Aspartate aminotransferase, mitochondrial
	3207/3228	MUC-5AC & Uncharacterized serine-rich protein		
	3262/3263	Probable aldo-keto reductase 2 & Aldo-keto reductase yakc		
**Putative chromosome 18**			7028	Bromodomain and WD repeat-containing protein 3
**Putative chromosome 19**	1276/1280	Probable quinate permease		
**Putative chromosome 20**			2269/2270	TCP-1-zeta
**Putative chromosome 21**	5744	Pneumococcal serine-rich repeat protein		

* Paralogous sequences from DBVPG 8058 that are located in chromosomes containing large translocations compared to CBS 7808. The paralogous sequences with orthologs in the same putative chromosome from the other *R. babjevae* strain are indicated in black.

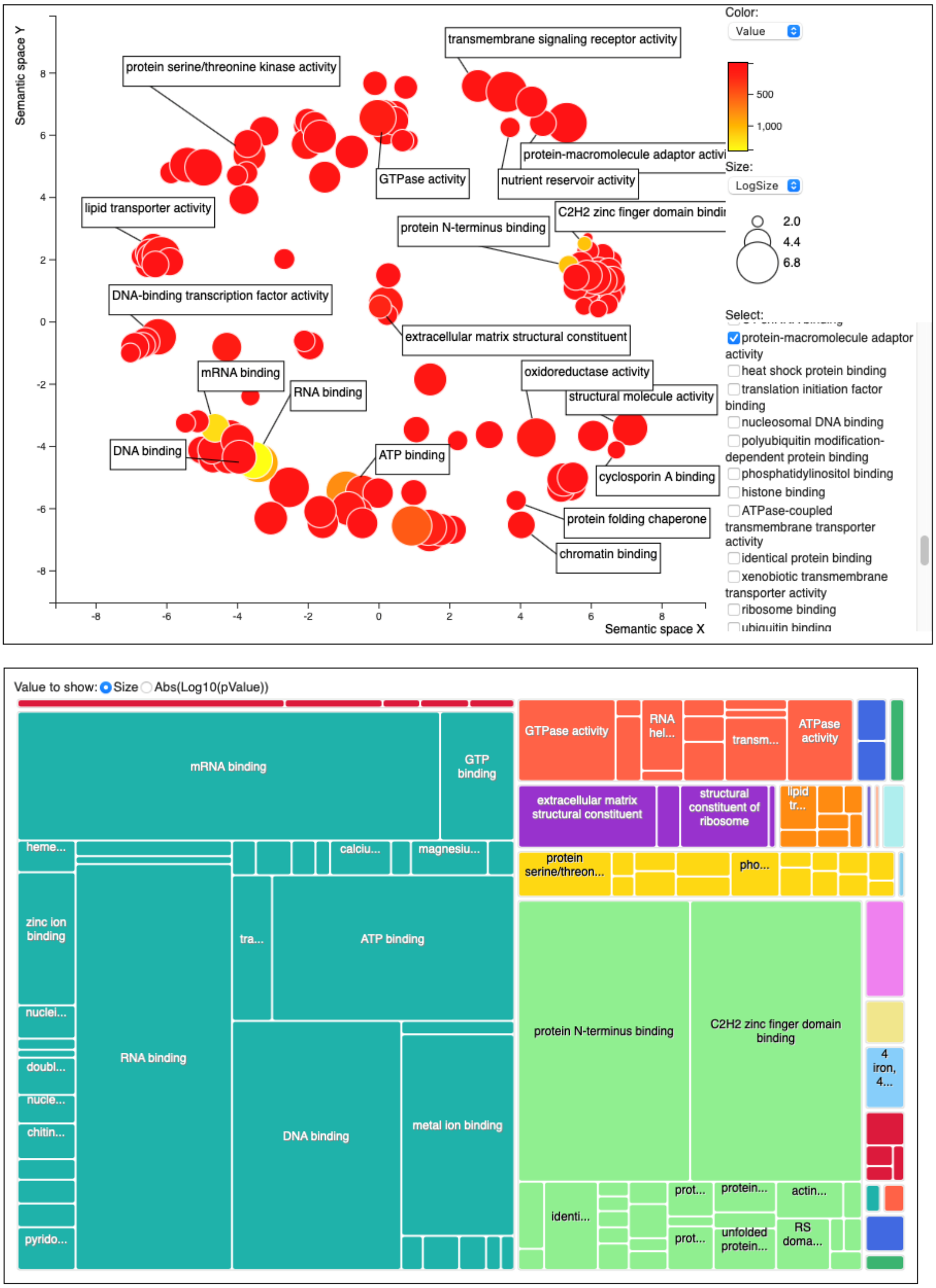

(a)

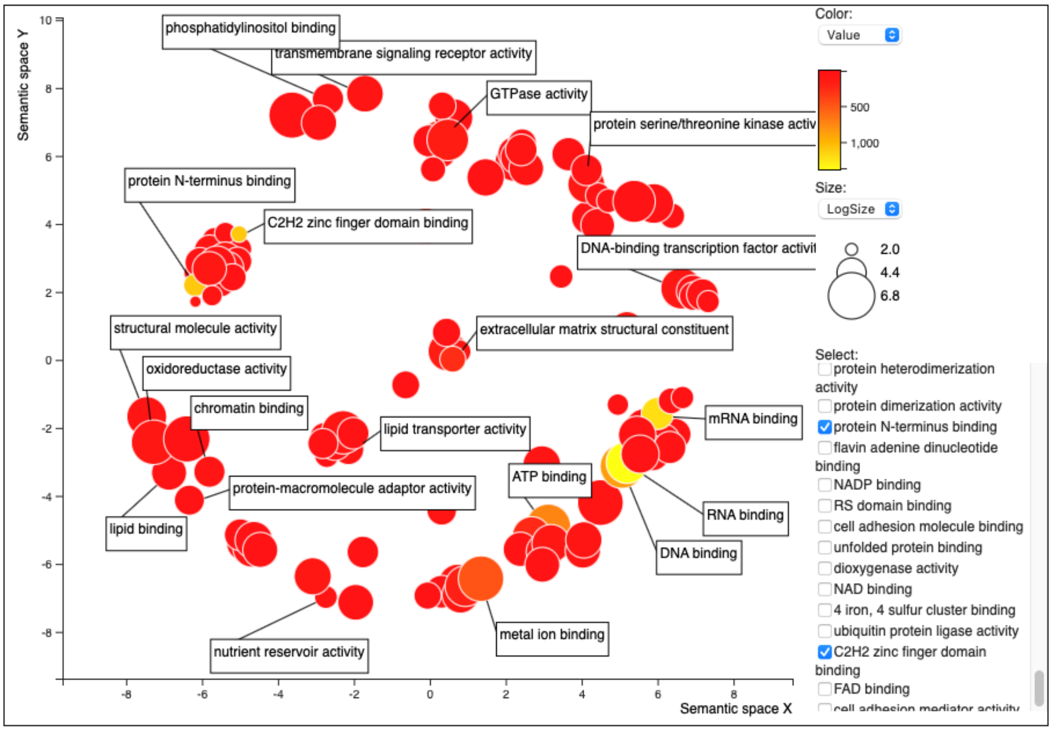

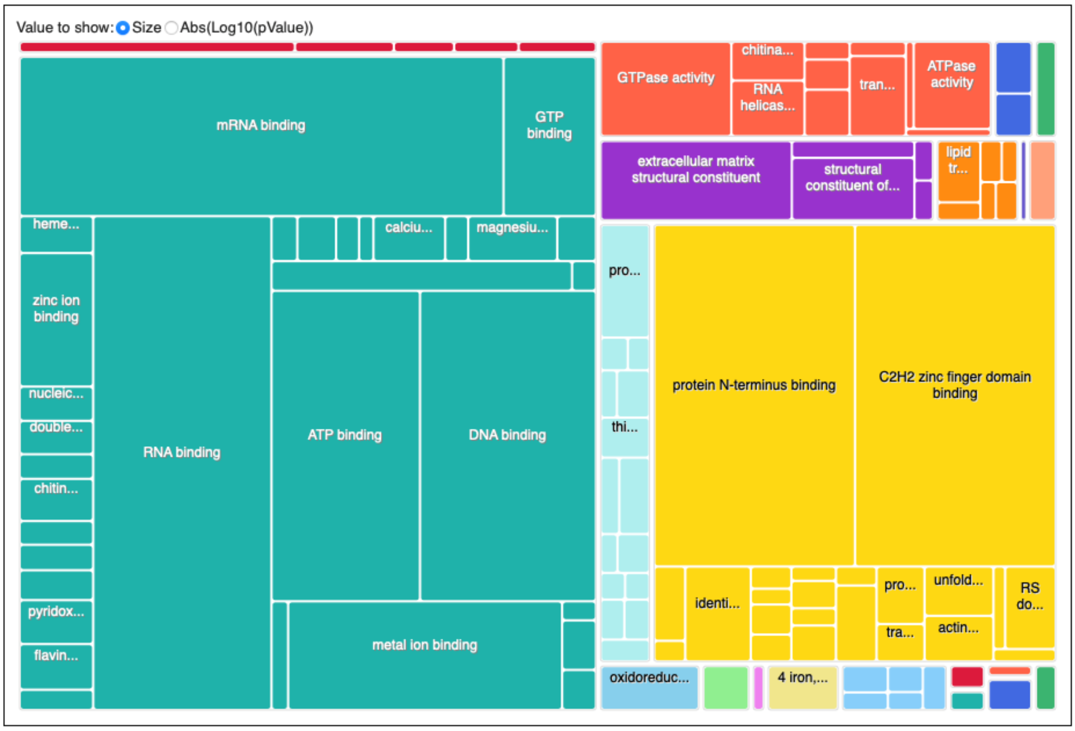

(b)

Supplementary Figure S1. Gene Ontology (GO) term summary related to the GO topic: molecular functions. (a) *Rhodotorula babjevae* CBS 7808; (b) *R. babjevae* DBVPG 8058.

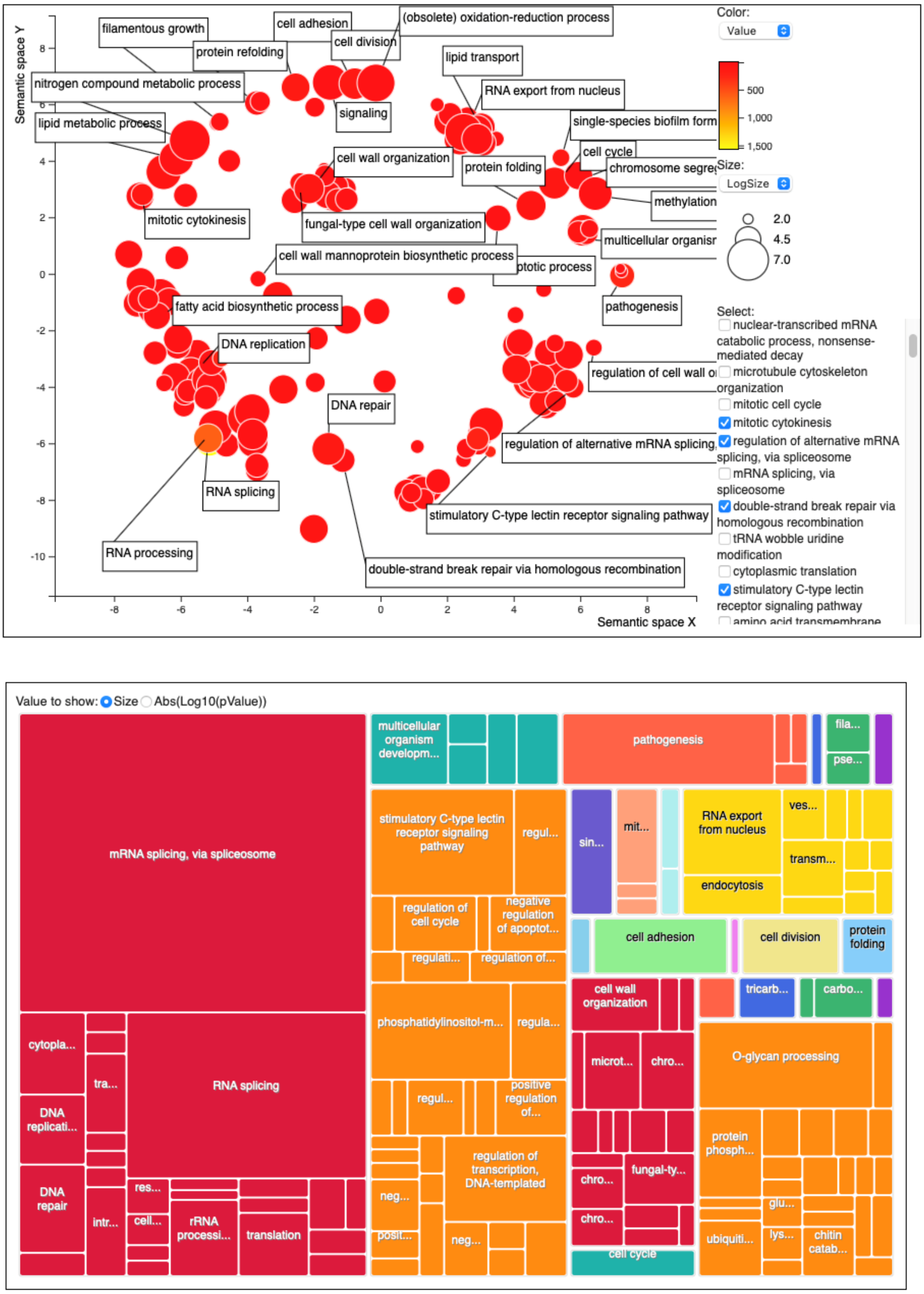

Semantic space Y
Semantic space X
filamentous growth
cell adhesion
(obsolete) oxidation-reduction process
cell division
protein refolding
nitrogen compound metabolic process
signaling
lipid transport
RNA export from nucleus
lipid metabolic process
single-species biofilm form
cell cycle
cell wall organization
protein folding
chromosome segre
methylation
mitotic cytokinesis
fungal-type cell wall organization
multicellular organisr
cell wall mannoprotein biosynthetic process
ptotic process
pathogenesis
fatty acid biosynthetic process
DNA replication
regulation of cell wall o
DNA repair
regulation of alternative mRNA splicing
RNA splicing
stimulatory C-type lectin receptor signaling pathway
RNA processing
double-strand break repair via homologous recombination
Color:
Value
500
1,000
1,500
Size:
LogSize
2.0
4.5
7.0
Select:
nuclear-transcribed mRNA catabolic process, nonsense-mediated decay
microtubule cytoskeleton organization
mitotic cell cycle
mitotic cytokinesis
regulation of alternative mRNA splicing, via spliceosome
mRNA splicing, via spliceosome
double-strand break repair via homologous recombination
tRNA wobble uridine modification
cytoplasmic translation
stimulatory C-type lectin receptor signaling pathway
Value to show: Size Abs(Log10(pValue))
mRNA splicing, via spliceosome
multicellular organism developm...
pathogenesis
fila...
pse...
stimulatory C-type lectin receptor signaling pathway
regul...
sin...
mit...
RNA export from nucleus
ves...
transm...
endocytosis
regulation of cell cycle
negative regulation of apoptot...
regulati...
regulation of...
cell adhesion
cell division
protein folding
cytopla...
tra...
RNA splicing
phosphatidylinositol-m...
regula...
cell wall organization
tricarb...
carbo...
DNA replicati...
regul...
positive regulation of...
microt...
chro...
O-glycan processing
DNA repair
regulation of transcription, DNA-templated
protein phosph...
res...
neg...
fungal-ty...
chro...
glu...
intr...
cell...
rRNA processi...
translation
posit...
neg...
chro...
ubiquiti...
lys...
chitin catab...
cell cycle

(a)

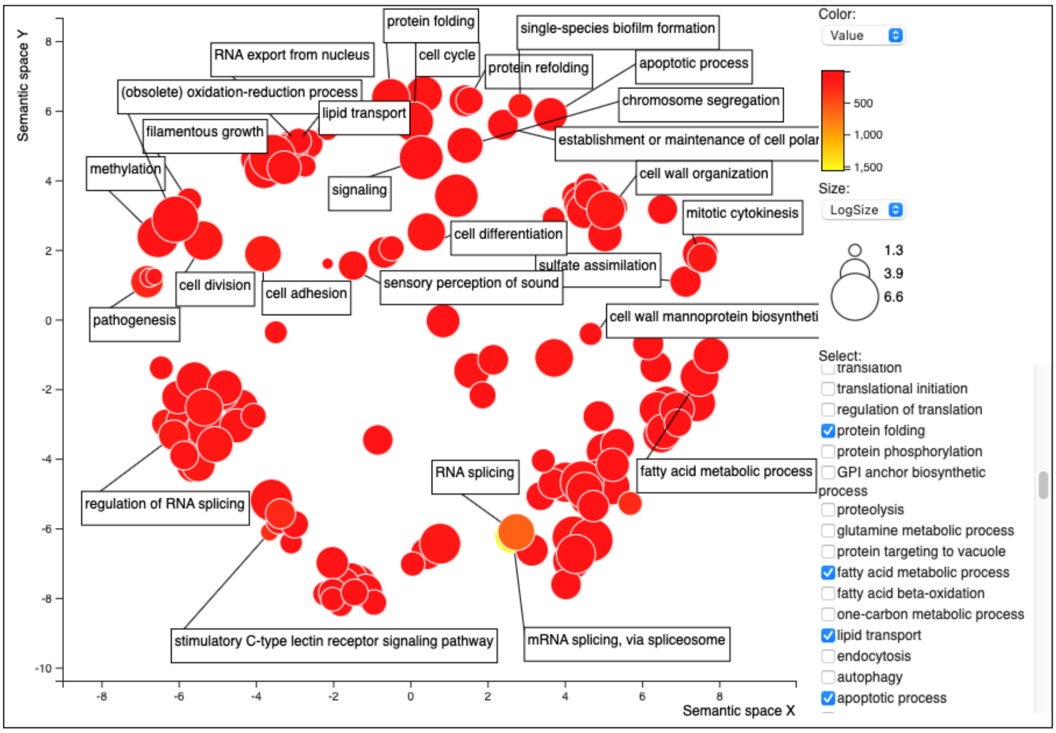

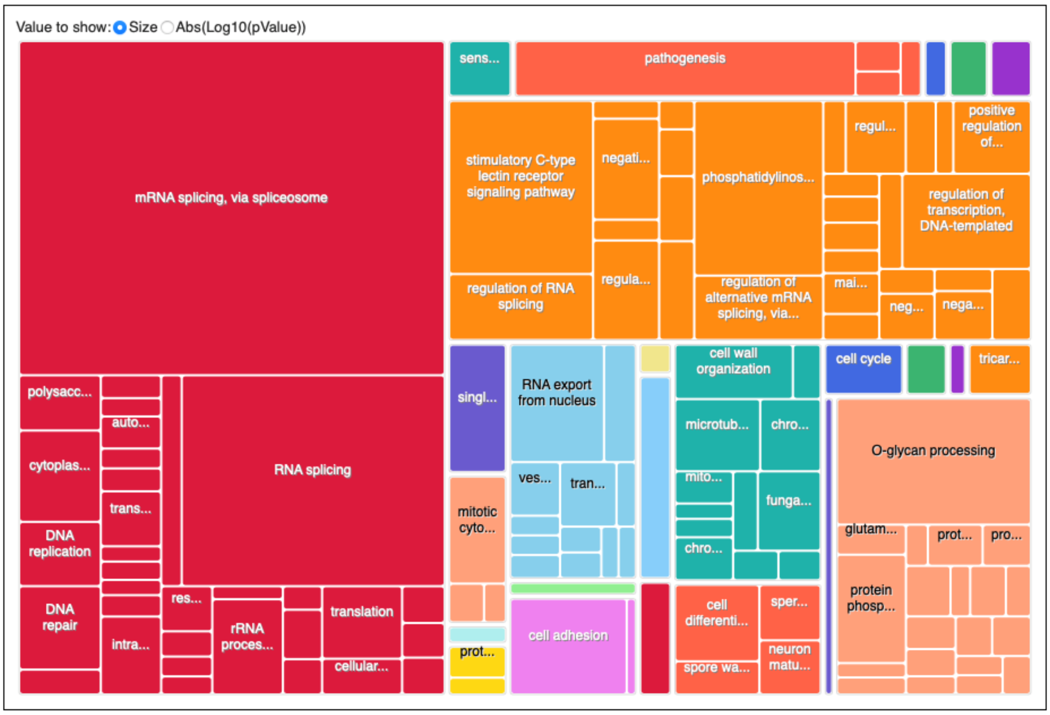

(b)

Supplementary Figure S2. Gene Ontology (GO) term summaries belonging to the GO topic: biological processes. (a) *Rhodotorula babjevae* CBS 7808; (b) *R. babjevae* DBVPG 8058.

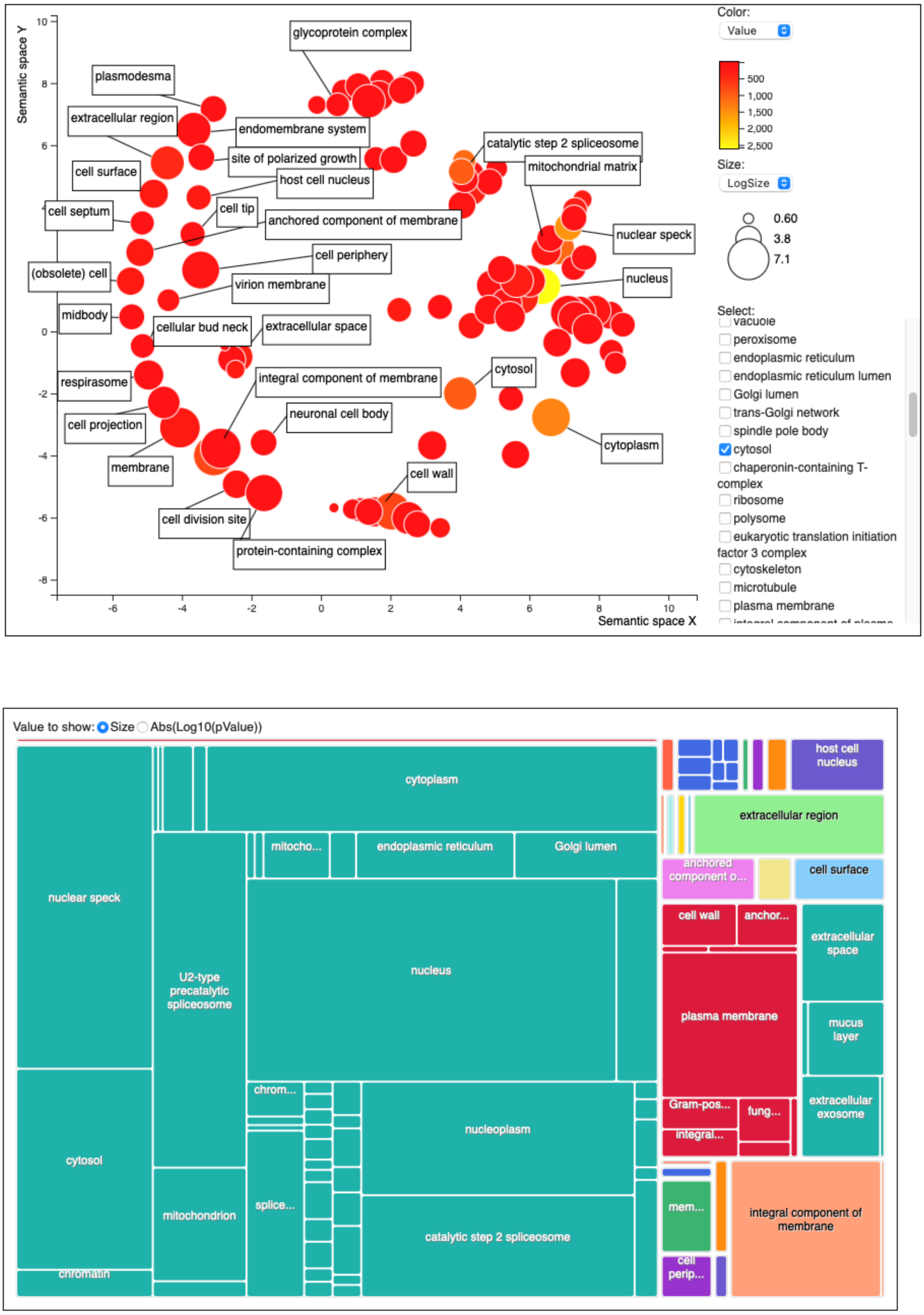

Semantic space Y
Semantic space X
glycoprotein complex
plasmodesma
extracellular region
endomembrane system
site of polarized growth
host cell nucleus
cell surface
cell septum
cell tip
anchored component of membrane
catalytic step 2 spliceosome
mitochondrial matrix
nuclear speck
cell periphery
(obsolete) cell
virion membrane
nucleus
midbody
cellular bud neck
extracellular space
respirasome
integral component of membrane
cytosol
cell projection
neuronal cell body
membrane
cytoplasm
cell wall
cell division site
protein-containing complex
Color:
Value
Size:
LogSize
Select:
peroxisome
endoplasmic reticulum
endoplasmic reticulum lumen
Golgi lumen
trans-Golgi network
spindle pole body
cytosol
chaperonin-containing T-complex
ribosome
polysome
eukaryotic translation initiation factor 3 complex
cytoskeleton
microtubule
plasma membrane
Value to show: Size Abs(Log10(pValue))
cytoplasm
nuclear speck
mitocho...
endoplasmic reticulum
Golgi lumen
U2-type precatalytic spliceosome
nucleus
chrom...
nucleoplasm
cytosol
mitochondrion
splice...
catalytic step 2 spliceosome
chromatin
host cell nucleus
extracellular region
anchored component o...
cell surface
cell wall
anchor...
extracellular space
plasma membrane
mucus layer
Gram-pos...
fung...
integral...
extracellular exosome
mem...
integral component of membrane
cell perip...

(a)

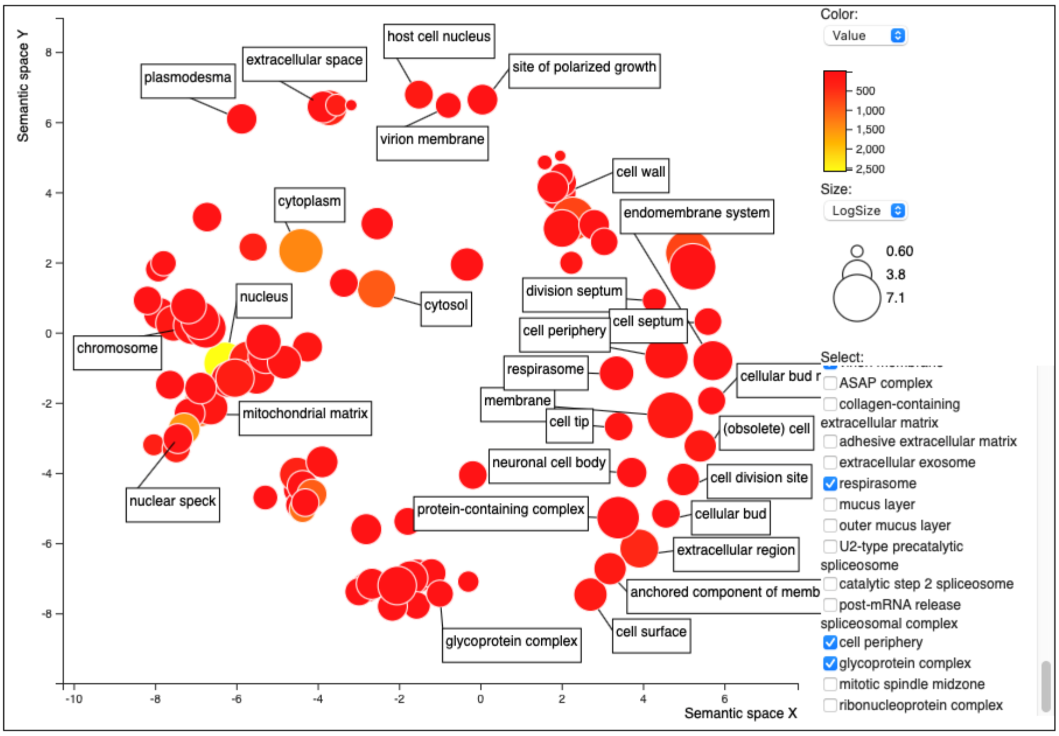

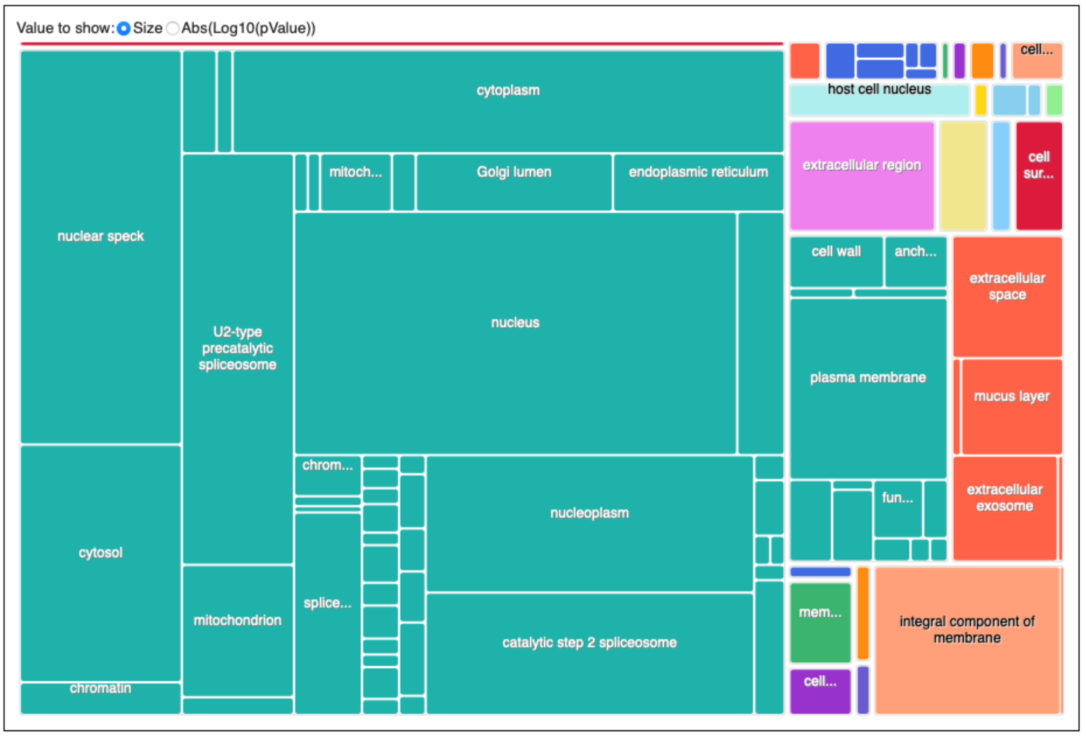

(b)

Supplementary Figure S3. Gene Ontology (GO) term summaries belonging to the GO topic: cellular components. (a) *Rhodotorula babjevae* CBS 7808; (b) *R. babjevae* DBVPG 8058.

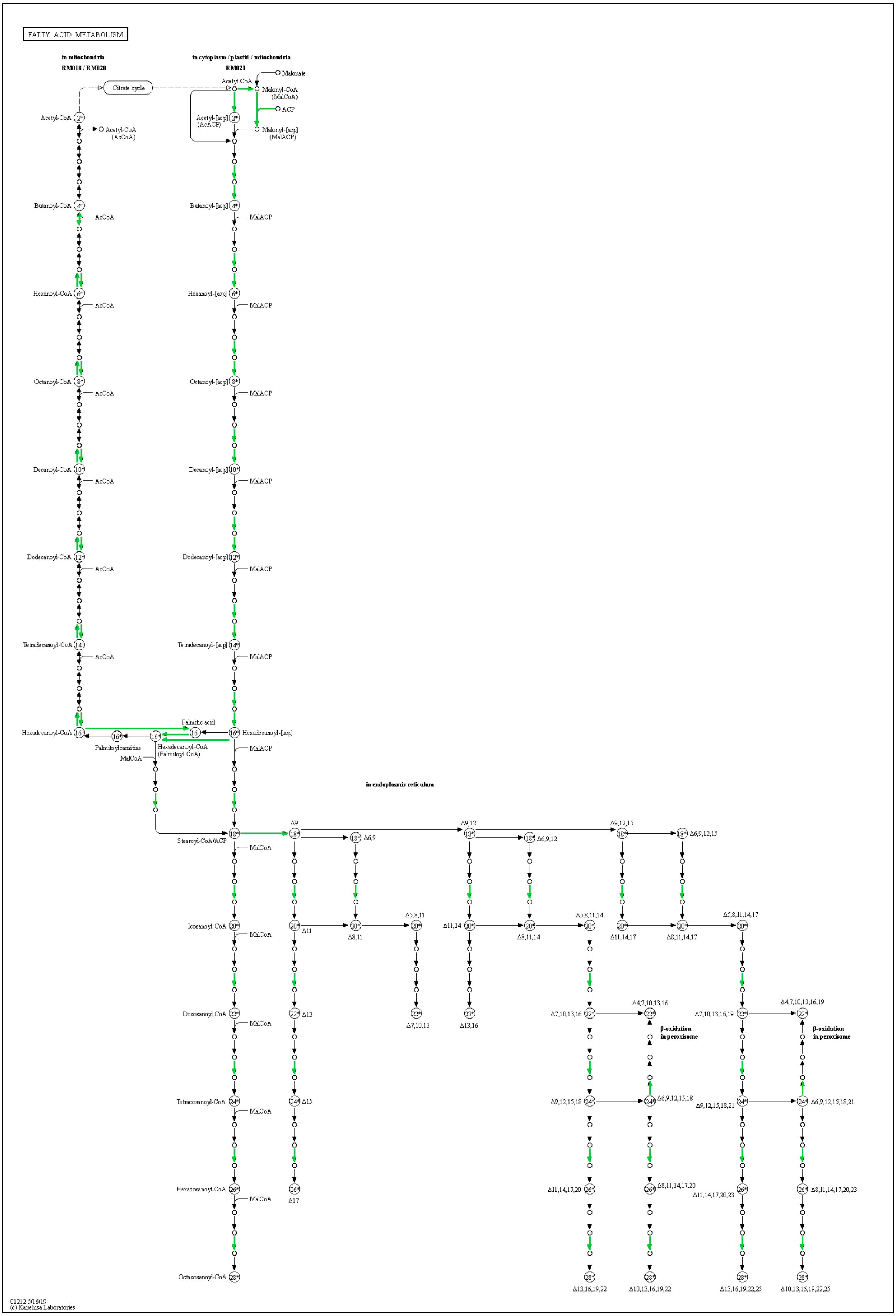
FATTY ACID METABOLISM
in mitochondria
RM018 / RM020
in cytoplasm / plastid / mitochondria
RM021
Citrate cycle
Acetyl-CoA
Malonate
Malonyl-CoA
(MalCoA)
ACP
Acetyl-[acp]
(AcACP)
Malonyl-[acp]
(MalACP)
Acetyl-CoA
(AcCoA)
Butanoyl-CoA
Butanoyl-[acp]
Hexanoyl-CoA
Hexanoyl-[acp]
Octanoyl-CoA
Octanoyl-[acp]
Decanoyl-CoA
Decanoyl-[acp]
Dodecanoyl-CoA
Dodecanoyl-[acp]
Tetradecanoyl-CoA
Tetradecanoyl-[acp]
Hexadecanoyl-CoA
Palmitic acid
Hexadecanoyl-[acp]
Palmitoylcarnitine
Hexadecanoyl-CoA
(Palmitoyl-CoA)
MalCoA
AcCoA
MalACP
in endoplasmic reticulum
Stearoyl-CoA/ACP
Icosanoyl-CoA
Docosanoyl-CoA
Tetracosanoyl-CoA
Hexacosanoyl-CoA
Octacosanoyl-CoA
β-oxidation
in peroxisome
01212 5/16/19
(c) Kanehisa Laboratories

(a)

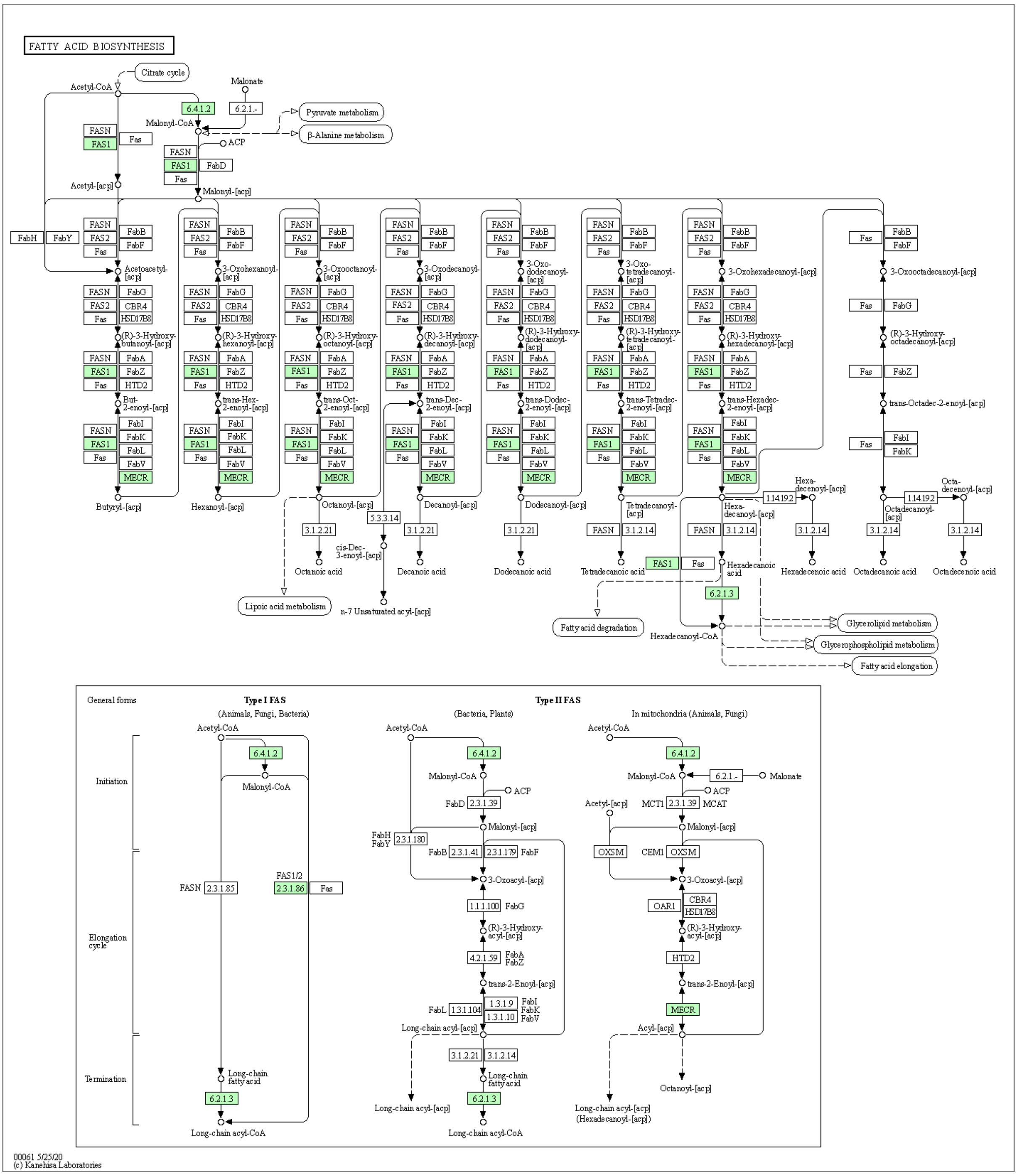

FATTY ACID BIOSYNTHESIS
Citrate cycle
Acetyl-CoA
Malonate
6.4.1.2
6.2.1.-
Pyruvate metabolism
β-Alanine metabolism
Malonyl-CoA
ACP
FASN
FAS1
Fas
FabD
Acetyl-[acp]
Malonyl-[acp]
FabH
FabY
FAS2
FabB
FabF
FabG
CBR4
HSD17B8
FabA
FabZ
HTD2
FabI
FabK
FabL
FabV
MECR
Acetoacetyl-[acp]
(R)-3-Hydroxy-butanoyl-[acp]
But-2-enoyl-[acp]
Butyryl-[acp]
3-Oxohexanoyl-[acp]
(R)-3-Hydroxy-hexanoyl-[acp]
trans-Hex-2-enoyl-[acp]
Hexanoyl-[acp]
3-Oxooctanoyl-[acp]
(R)-3-Hydroxy-octanoyl-[acp]
trans-Oct-2-enoyl-[acp]
Octanoyl-[acp]
3-Oxodecanoyl-[acp]
(R)-3-Hydroxy-decanoyl-[acp]
trans-Dec-2-enoyl-[acp]
Decanoyl-[acp]
3-Oxo-dodecanoyl-[acp]
(R)-3-Hydroxy-dodecanoyl-[acp]
trans-Dodec-2-enoyl-[acp]
Dodecanoyl-[acp]
3-Oxo-tetradecanoyl-[acp]
(R)-3-Hydroxy-tetradecanoyl-[acp]
trans-Tetradec-2-enoyl-[acp]
Tetradecanoyl-[acp]
3-Oxohexadecanoyl-[acp]
(R)-3-Hydroxy-hexadecanoyl-[acp]
trans-Hexadec-2-enoyl-[acp]
Hexadecanoyl-[acp]
3-Oxooctadecanoyl-[acp]
(R)-3-Hydroxy-octadecanoyl-[acp]
trans-Octadec-2-enoyl-[acp]
Octadecanoyl-[acp]
Hexadecenoyl-[acp]
Octadecenoyl-[acp]
1.14.19.2
3.1.2.21
3.1.2.14
5.3.3.14
cis-Dec-3-enoyl-[acp]
Octanoic acid
Decanoic acid
Dodecanoic acid
Tetradecanoic acid
Hexadecanoic acid
Hexadecenoic acid
Octadecanoic acid
Octadecenoic acid
Lipoic acid metabolism
n-7 Unsaturated acyl-[acp]
6.2.1.3
Fatty acid degradation
Hexadecanoyl-CoA
Glycerolipid metabolism
Glycerophospholipid metabolism
Fatty acid elongation
General forms
Type I FAS
(Animals, Fungi, Bacteria)
Type II FAS
(Bacteria, Plants)
In mitochondria (Animals, Fungi)
Initiation
Elongation cycle
Termination
2.3.1.85
FAS1/2
2.3.1.86
Long-chain fatty acid
Long-chain acyl-CoA
FabD
2.3.1.39
FabH
FabY
2.3.1.180
2.3.1.41
2.3.1.179
3-Oxoacyl-[acp]
1.1.1.100
(R)-3-Hydroxy-acyl-[acp]
4.2.1.59
trans-2-Enoyl-[acp]
1.3.1.104
1.3.1.9
1.3.1.10
Long-chain acyl-[acp]
MCT1
MCAT
OXSM
CEM1
OAR1
Acyl-[acp]
Octanoyl-[acp]
Long-chain acyl-[acp]
(Hexadecanoyl-[acp])
00061 5/25/20
(c) Kanehisa Laboratories

(b)

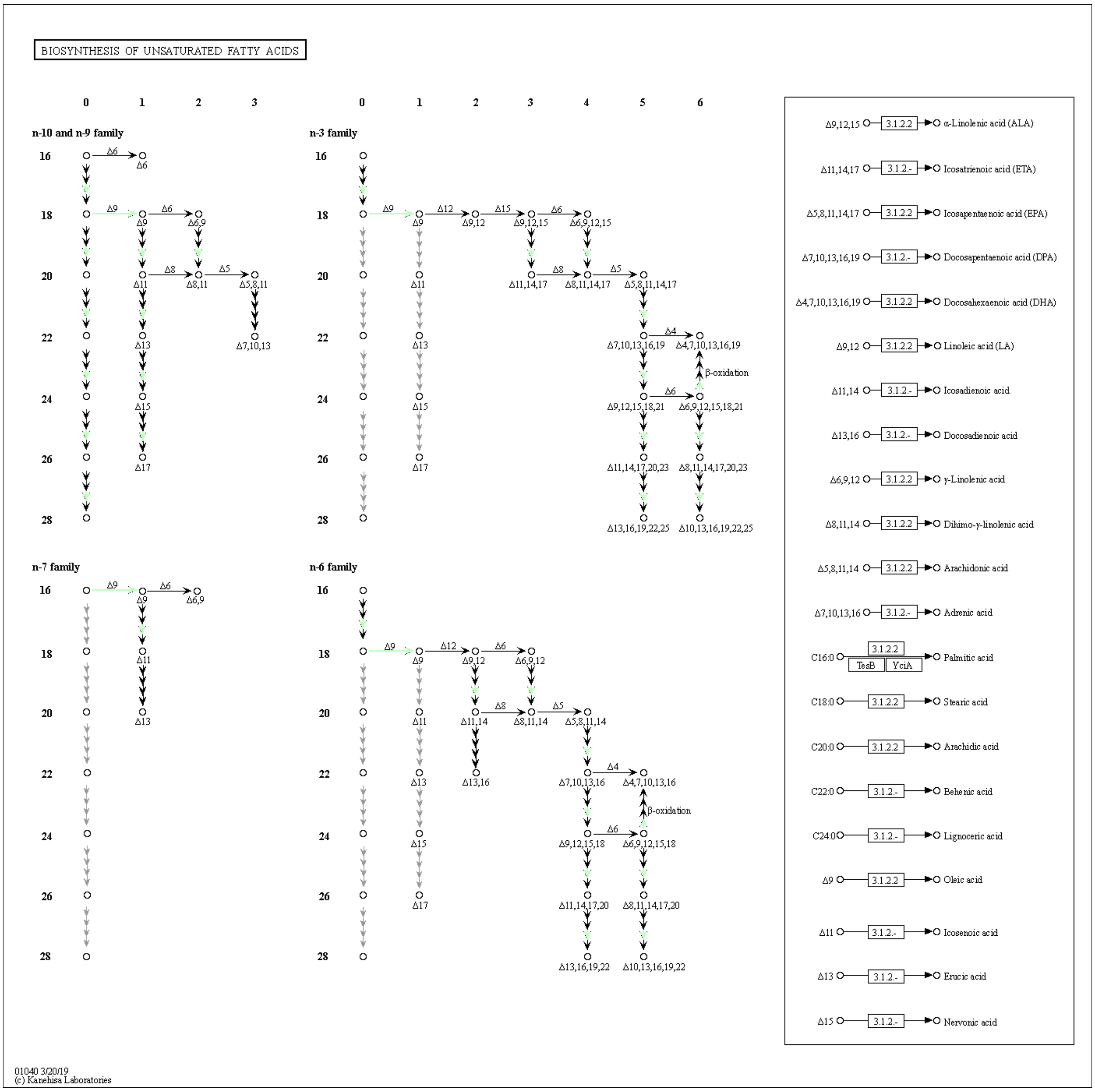

BIOSYNTHESIS OF UNSATURATED FATTY ACIDS
n-10 and n-9 family
n-3 family
n-7 family
n-6 family
0 1 2 3 4 5 6
16 18 20 22 24 26 28
Δ6
Δ9
Δ11
Δ13
Δ15
Δ17
Δ6,9
Δ8,11
Δ5,8,11
Δ7,10,13
Δ8
Δ5
Δ12
Δ4
Δ9,12
Δ9,12,15
Δ6,9,12,15
Δ11,14,17
Δ8,11,14,17
Δ5,8,11,14,17
Δ7,10,13,16,19
Δ4,7,10,13,16,19
β-oxidation
Δ9,12,15,18,21
Δ6,9,12,15,18,21
Δ11,14,17,20,23
Δ8,11,14,17,20,23
Δ13,16,19,22,25
Δ10,13,16,19,22,25
Δ6,9,12
Δ11,14
Δ8,11,14
Δ5,8,11,14
Δ13,16
Δ7,10,13,16
Δ4,7,10,13,16
Δ9,12,15,18
Δ6,9,12,15,18
Δ11,14,17,20
Δ8,11,14,17,20
Δ13,16,19,22
Δ10,13,16,19,22
Δ9,12,15 3.1.2.2 α-Linolenic acid (ALA)
Δ11,14,17 3.1.2.- Icosatrienoic acid (ETA)
Δ5,8,11,14,17 3.1.2.2 Icosapentaenoic acid (EPA)
Δ7,10,13,16,19 3.1.2.- Docosapentaenoic acid (DPA)
Δ4,7,10,13,16,19 3.1.2.2 Docosahexaenoic acid (DHA)
Δ9,12 3.1.2.2 Linoleic acid (LA)
Δ11,14 3.1.2.- Icosadienoic acid
Δ13,16 3.1.2.- Docosadienoic acid
Δ6,9,12 3.1.2.2 γ-Linolenic acid
Δ8,11,14 3.1.2.2 Dihimo-γ-linolenic acid
Δ5,8,11,14 3.1.2.2 Arachidonic acid
Δ7,10,13,16 3.1.2.- Adrenic acid
C16:0 3.1.2.2 TesB YciA Palmitic acid
C18:0 3.1.2.2 Stearic acid
C20:0 3.1.2.2 Arachidic acid
C22:0 3.1.2.- Behenic acid
C24:0 3.1.2.- Lignoceric acid
Δ9 3.1.2.2 Oleic acid
Δ11 3.1.2.- Icosenoic acid
Δ13 3.1.2.- Erucic acid
Δ15 3.1.2.- Nervonic acid
01040 3/20/19
(c) Kanehisa Laboratories

(c)

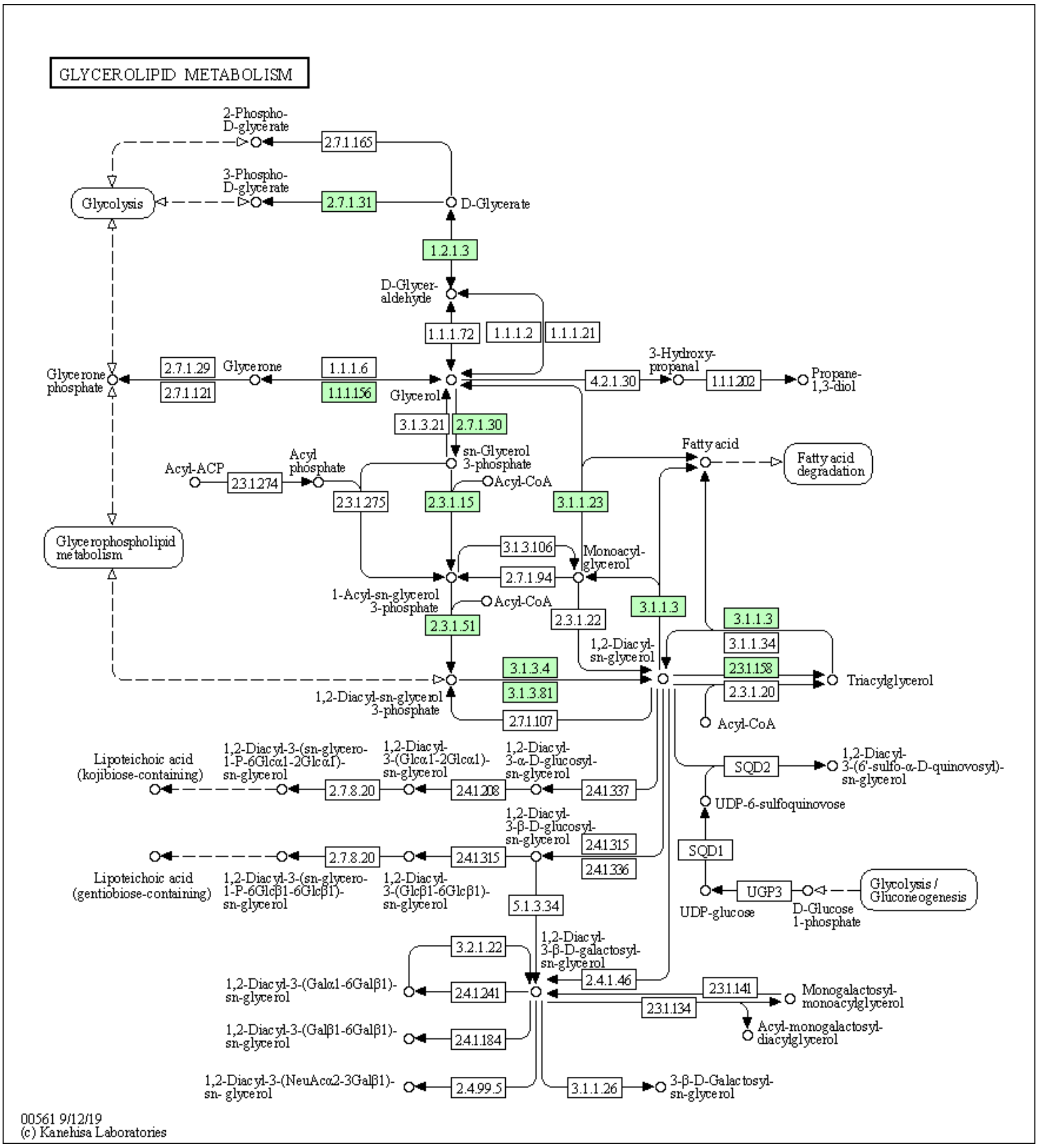
GLYCEROLIPID METABOLISM
2-Phospho-D-glycerate
3-Phospho-D-glycerate
Glycolysis
D-Glycerate
D-Glyceraldehyde
Glycerone phosphate
Glycerone
Glycerol
3-Hydroxy-propanal
Propane-1,3-diol
sn-Glycerol 3-phosphate
Acyl-ACP
Acyl phosphate
Acyl-CoA
Fatty acid
Fatty acid degradation
Glycerophospholipid metabolism
Monoacylglycerol
1-Acyl-sn-glycerol 3-phosphate
1,2-Diacyl-sn-glycerol
Triacylglycerol
1,2-Diacyl-sn-glycerol 3-phosphate
1,2-Diacyl-3-(6'-sulfo-α-D-quinovosyl)-sn-glycerol
UDP-6-sulfoquinovose
UDP-glucose
D-Glucose 1-phosphate
Glycolysis / Gluconeogenesis
Lipoteichoic acid (kojibiose-containing)
Lipoteichoic acid (gentiobiose-containing)
1,2-Diacyl-3-β-D-galactosyl-sn-glycerol
Monogalactosyl-monoacylglycerol
Acyl-monogalactosyl-diacylglycerol
3-β-D-Galactosyl-sn-glycerol
00561 9/12/19
(c) Kanehisa Laboratories

(d)

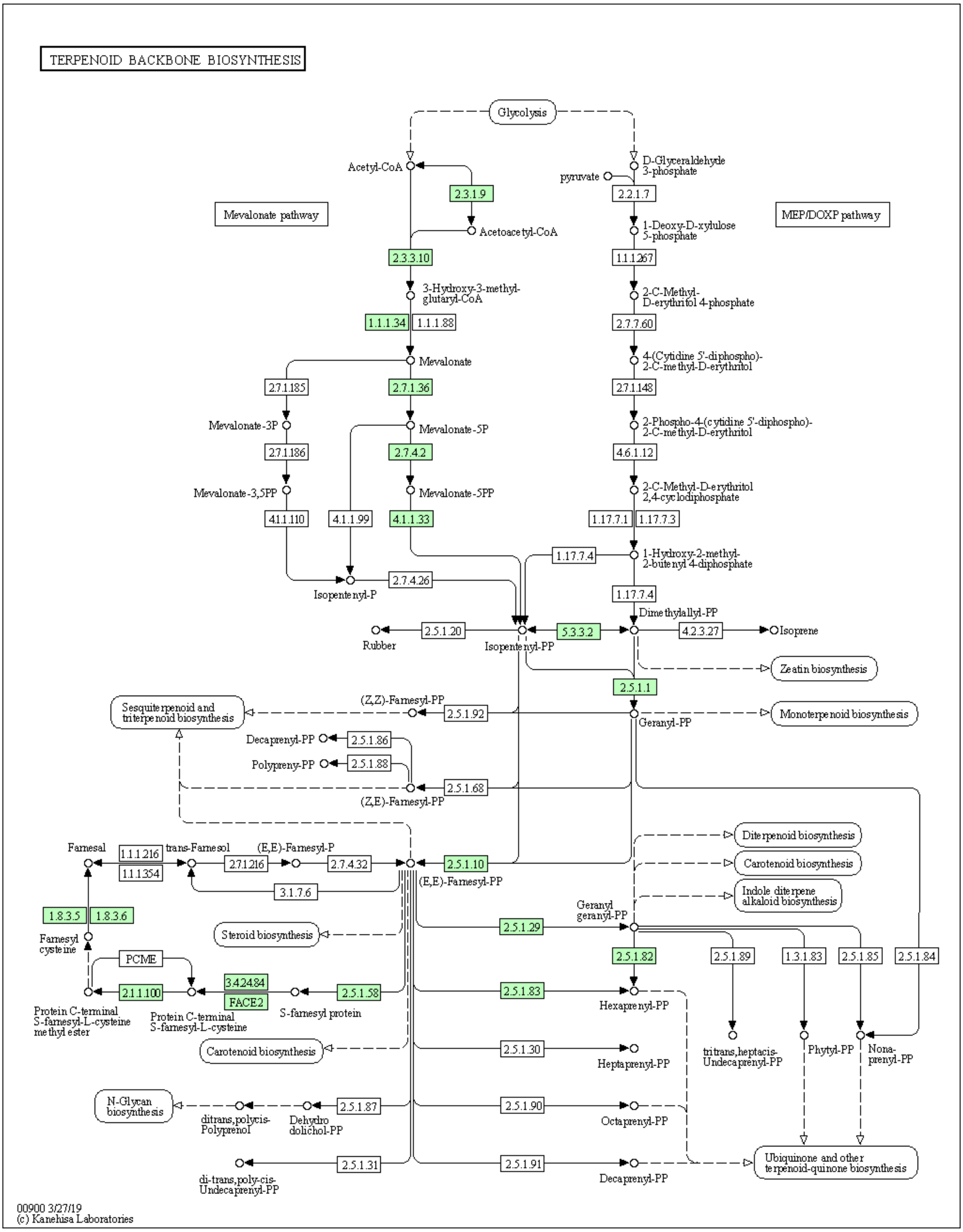
TERPENOID BACKBONE BIOSYNTHESIS
Glycolysis
Acetyl-CoA
D-Glyceraldehyde 3-phosphate
pyruvate
2.3.1.9
2.2.1.7
Mevalonate pathway
MEP/DOXP pathway
Acetoacetyl-CoA
1-Deoxy-D-xylulose 5-phosphate
2.3.3.10
1.1.1.267
3-Hydroxy-3-methyl-glutaryl-CoA
2-C-Methyl-D-erythritol 4-phosphate
1.1.1.34
1.1.1.88
2.7.7.60
Mevalonate
4-(Cytidine 5'-diphospho)-2-C-methyl-D-erythritol
2.7.1.185
2.7.1.36
2.7.1.148
Mevalonate-3P
Mevalonate-5P
2-Phospho-4-(cytidine 5'-diphospho)-2-C-methyl-D-erythritol
2.7.1.186
2.7.4.2
4.6.1.12
Mevalonate-3,5PP
Mevalonate-5PP
2-C-Methyl-D-erythritol 2,4-cyclodiphosphate
4.1.1.110
4.1.1.99
4.1.1.33
1.17.7.1
1.17.7.3
1.17.7.4
1-Hydroxy-2-methyl-2-butenyl 4-diphosphate
2.7.4.26
Isopentenyl-P
1.17.7.4
Dimethylallyl-PP
Rubber
2.5.1.20
5.3.3.2
4.2.3.27
Isoprene
Isopentenyl-PP
Zeatin biosynthesis
2.5.1.1
Sesquiterpenoid and triterpenoid biosynthesis
(Z,Z)-Farnesyl-PP
2.5.1.92
Geranyl-PP
Monoterpenoid biosynthesis
Decaprenyl-PP
2.5.1.86
Polyprenyl-PP
2.5.1.88
2.5.1.68
(Z,E)-Farnesyl-PP
Diterpenoid biosynthesis
Farnesal
1.1.1.216
trans-Farnesol
(E,E)-Farnesyl-P
1.1.1.354
2.7.1.216
2.7.4.32
2.5.1.10
Carotenoid biosynthesis
3.1.7.6
(E,E)-Farnesyl-PP
Indole diterpene alkaloid biosynthesis
1.8.3.5
1.8.3.6
Geranyl geranyl-PP
Steroid biosynthesis
2.5.1.29
Farnesyl cysteine
PCME
2.5.1.82
2.5.1.89
1.3.1.83
2.5.1.85
2.5.1.84
3.4.24.84
2.1.1.100
2.5.1.58
FACE2
2.5.1.83
Hexaprenyl-PP
Protein C-terminal S-farnesyl-L-cysteine methyl ester
Protein C-terminal S-farnesyl-L-cysteine
S-farnesyl protein
tritrans,heptacis-Undecaprenyl-PP
Phytyl-PP
Nona-prenyl-PP
Carotenoid biosynthesis
2.5.1.30
Heptaprenyl-PP
N-Glycan biosynthesis
2.5.1.87
2.5.1.90
ditrans,polycis-Polyprenol
Dehydro dolichol-PP
Octaprenyl-PP
Ubiquinone and other terpenoid-quinone biosynthesis
2.5.1.31
2.5.1.91
di-trans,poly-cis-Undecaprenyl-PP
Decaprenyl-PP
00900 3/27/19
(c) Kanehisa Laboratories

(e)

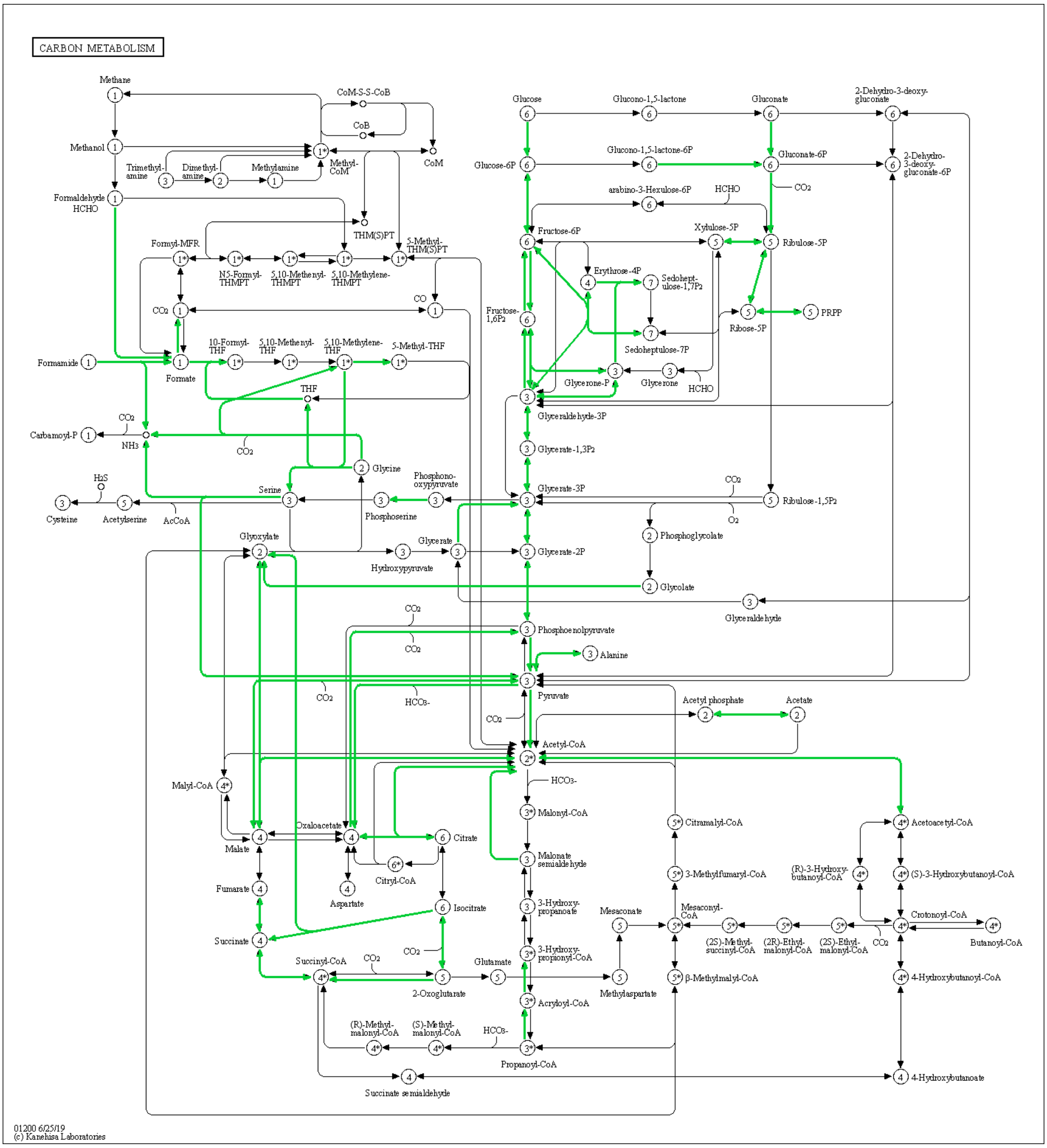

(f)

Supplementary Figure S4. Examples of lipid metabolism pathways in *Rhodotorula babjevae* CBS 7808 reconstructed by KEGG Mapper. The annotated transcripts with affiliated KEGG Orthology (KO) identifiers involved in each metabolic pathway are colored in green. (a) Fatty acid metabolism pathways; (b) biosynthesis of fatty acids; (c) biosynthesis of unsaturated fatty acids; (d) glycerolipid methabolism; (e) terpenoid backbone biosynthesis; (f) Carbon metabolism.

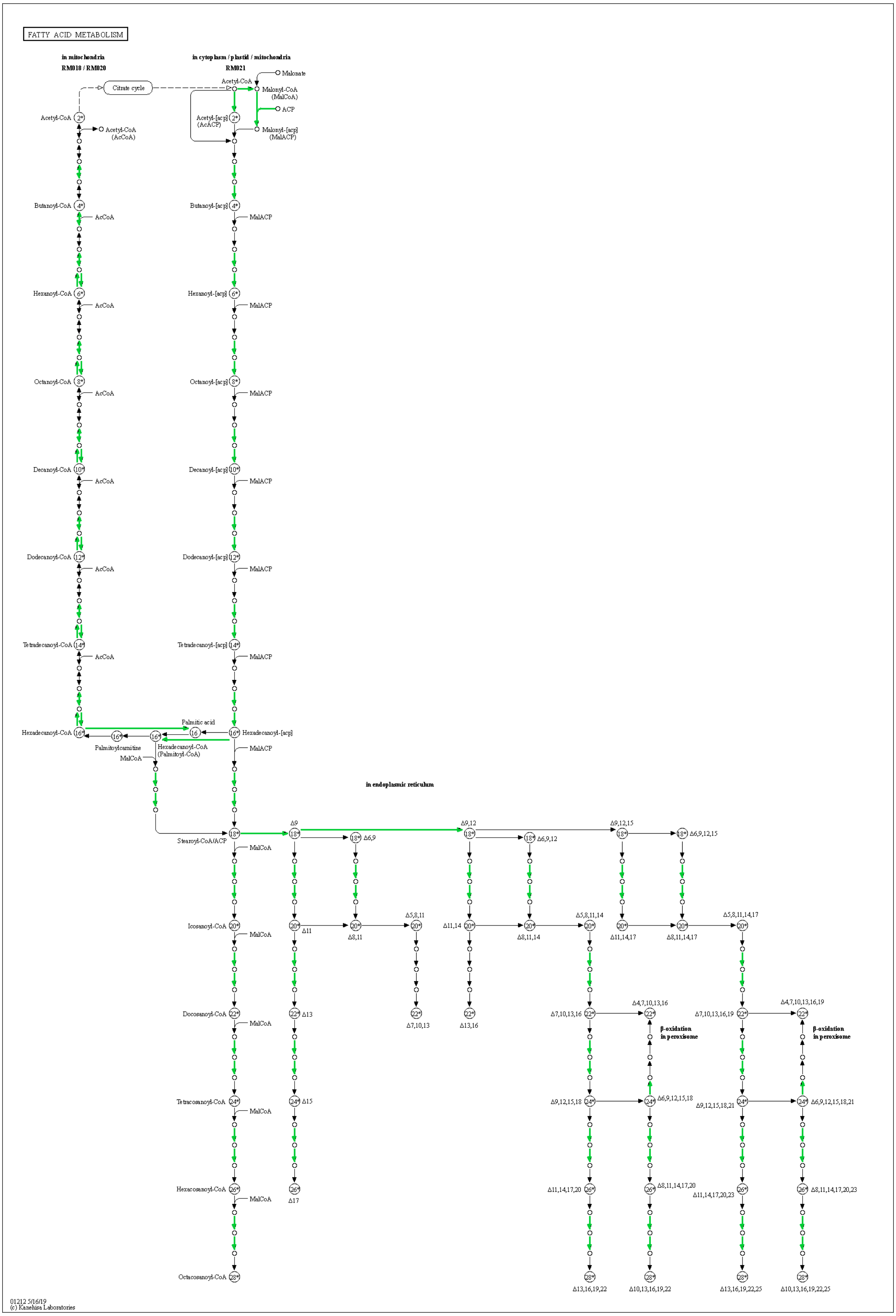

FATTY ACID METABOLISM
in mitochondria
RM018 / RM020
in cytoplasm / plastid / mitochondria
RM021
Citrate cycle
Acetyl-CoA
Malonate
Malonyl-CoA
(MalCoA)
ACP
Acetyl-[acp]
(AcACP)
Malonyl-[acp]
(MalACP)
Acetyl-CoA
(AcCoA)
Butanoyl-CoA
Butanoyl-[acp]
Hexanoyl-CoA
Hexanoyl-[acp]
Octanoyl-CoA
Octanoyl-[acp]
Decanoyl-CoA
Decanoyl-[acp]
Dodecanoyl-CoA
Dodecanoyl-[acp]
Tetradecanoyl-CoA
Tetradecanoyl-[acp]
Hexadecanoyl-CoA
Palmitic acid
Hexadecanoyl-[acp]
Palmitoylcarnitine
Hexadecanoyl-CoA
(Palmitoyl-CoA)
in endoplasmic reticulum
Stearoyl-CoA/ACP
Icosanoyl-CoA
Docosanoyl-CoA
Tetracosanoyl-CoA
Hexacosanoyl-CoA
Octacosanoyl-CoA
β-oxidation
in peroxisome
01212 5/16/19
(c) Kanehisa Laboratories

(a)

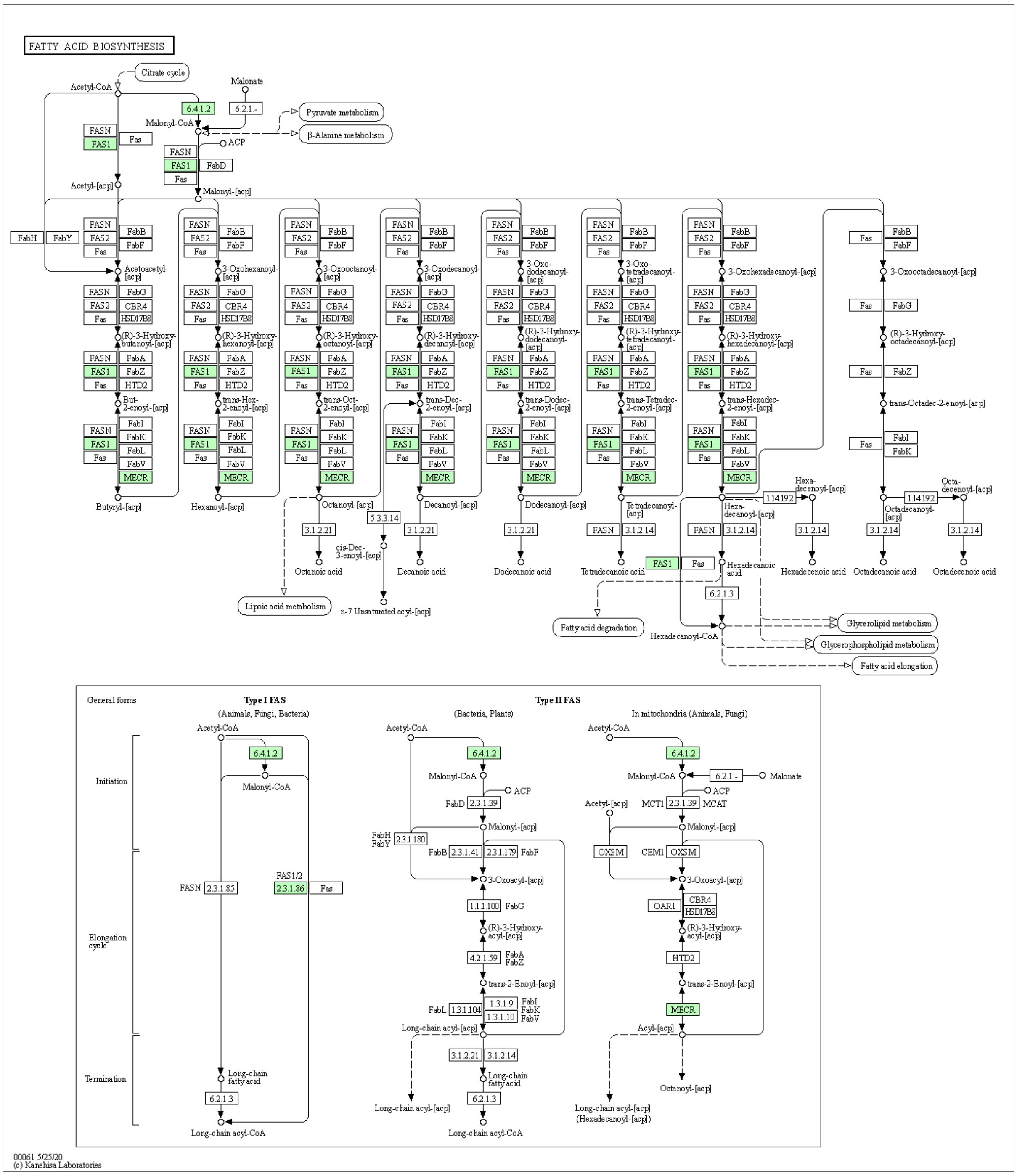
FATTY ACID BIOSYNTHESIS
Citrate cycle
Acetyl-CoA
Malonate
6.4.1.2
6.2.1.-
Pyruvate metabolism
β-Alanine metabolism
Malonyl-CoA
ACP
FASN
FAS1
Fas
FabD
Acetyl-[acp]
Malonyl-[acp]
FabH
FabY
FAS2
FabB
FabF
FabG
CBR4
HSD17B8
FabA
FabZ
HTD2
FabI
FabK
FabL
FabV
MECR
Acetoacetyl-[acp]
(R)-3-Hydroxy-butanoyl-[acp]
But-2-enoyl-[acp]
Butyryl-[acp]
3-Oxohexanoyl-[acp]
(R)-3-Hydroxy-hexanoyl-[acp]
trans-Hex-2-enoyl-[acp]
Hexanoyl-[acp]
3-Oxooctanoyl-[acp]
(R)-3-Hydroxy-octanoyl-[acp]
trans-Oct-2-enoyl-[acp]
Octanoyl-[acp]
3-Oxodecanoyl-[acp]
(R)-3-Hydroxy-decanoyl-[acp]
trans-Dec-2-enoyl-[acp]
Decanoyl-[acp]
3-Oxo-dodecanoyl-[acp]
(R)-3-Hydroxy-dodecanoyl-[acp]
trans-Dodec-2-enoyl-[acp]
Dodecanoyl-[acp]
3-Oxo-tetradecanoyl-[acp]
(R)-3-Hydroxy-tetradecanoyl-[acp]
trans-Tetradec-2-enoyl-[acp]
Tetradecanoyl-[acp]
3-Oxohexadecanoyl-[acp]
(R)-3-Hydroxy-hexadecanoyl-[acp]
trans-Hexadec-2-enoyl-[acp]
Hexa-decanoyl-[acp]
3-Oxooctadecanoyl-[acp]
(R)-3-Hydroxy-octadecanoyl-[acp]
trans-Octadec-2-enoyl-[acp]
Octadecanoyl-[acp]
1.14.19.2
Hexa-decenoyl-[acp]
Octa-decenoyl-[acp]
3.1.2.21
3.1.2.14
5.3.3.14
cis-Dec-3-enoyl-[acp]
Octanoic acid
Decanoic acid
Dodecanoic acid
Tetradecanoic acid
Hexadecanoic acid
Hexadecenoic acid
Octadecanoic acid
Octadecenoic acid
n-7 Unsaturated acyl-[acp]
Lipoic acid metabolism
6.2.1.3
Fatty acid degradation
Hexadecanoyl-CoA
Glycerolipid metabolism
Glycerophospholipid metabolism
Fatty acid elongation
General forms
Type I FAS
(Animals, Fungi, Bacteria)
Type II FAS
(Bacteria, Plants)
In mitochondria (Animals, Fungi)
Initiation
Elongation cycle
Termination
2.3.1.85
FAS1/2
2.3.1.86
Long-chain fatty acid
Long-chain acyl-CoA
2.3.1.39
2.3.1.180
2.3.1.41
2.3.1.179
3-Oxoacyl-[acp]
1.1.1.100
(R)-3-Hydroxy-acyl-[acp]
4.2.1.59
trans-2-Enoyl-[acp]
1.3.1.9
1.3.1.104
1.3.1.10
Long-chain acyl-[acp]
MCT1
MCAT
CEM1
OXSM
OAR1
Acyl-[acp]
Octanoyl-[acp]
Long-chain acyl-[acp] (Hexadecanoyl-[acp])
00061 5/25/20
(c) Kanehisa Laboratories

(b)

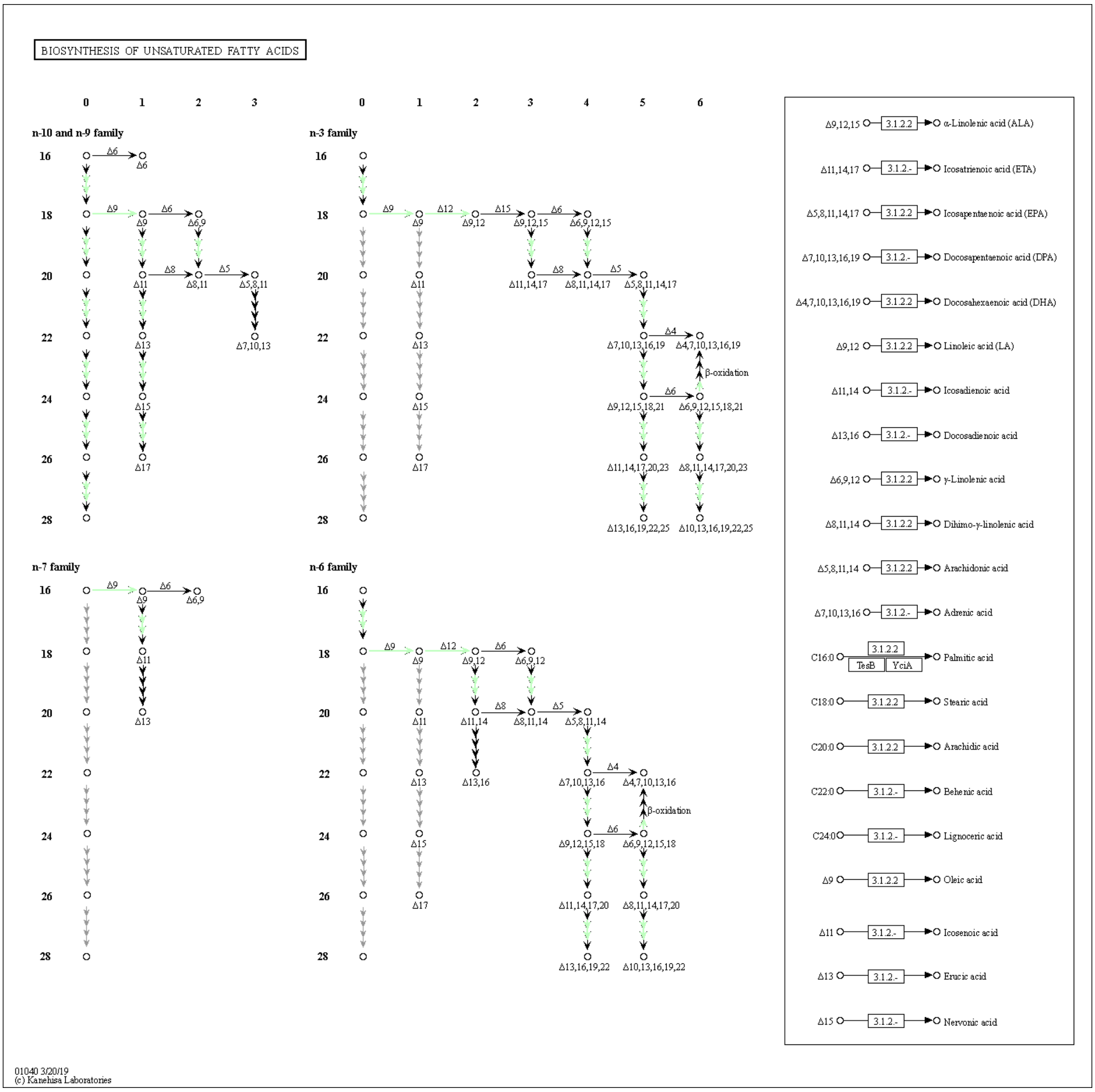

BIOSYNTHESIS OF UNSATURATED FATTY ACIDS
n-10 and n-9 family
n-3 family
n-7 family
n-6 family
β-oxidation
Δ9,12,15 3.1.2.2 α-Linolenic acid (ALA)
Δ11,14,17 3.1.2.- Icosatrienoic acid (ETA)
Δ5,8,11,14,17 3.1.2.2 Icosapentaenoic acid (EPA)
Δ7,10,13,16,19 3.1.2.- Docosapentaenoic acid (DPA)
Δ4,7,10,13,16,19 3.1.2.2 Docosahexaenoic acid (DHA)
Δ9,12 3.1.2.2 Linoleic acid (LA)
Δ11,14 3.1.2.- Icosadienoic acid
Δ13,16 3.1.2.- Docosadienoic acid
Δ6,9,12 3.1.2.2 γ-Linolenic acid
Δ8,11,14 3.1.2.2 Dihimo-γ-linolenic acid
Δ5,8,11,14 3.1.2.2 Arachidonic acid
Δ7,10,13,16 3.1.2.- Adrenic acid
C16:0 3.1.2.2 TesB YciA Palmitic acid
C18:0 3.1.2.2 Stearic acid
C20:0 3.1.2.2 Arachidic acid
C22:0 3.1.2.- Behenic acid
C24:0 3.1.2.- Lignoceric acid
Δ9 3.1.2.2 Oleic acid
Δ11 3.1.2.- Icosenoic acid
Δ13 3.1.2.- Erucic acid
Δ15 3.1.2.- Nervonic acid
01040 3/20/19
(c) Kanehisa Laboratories

(c)

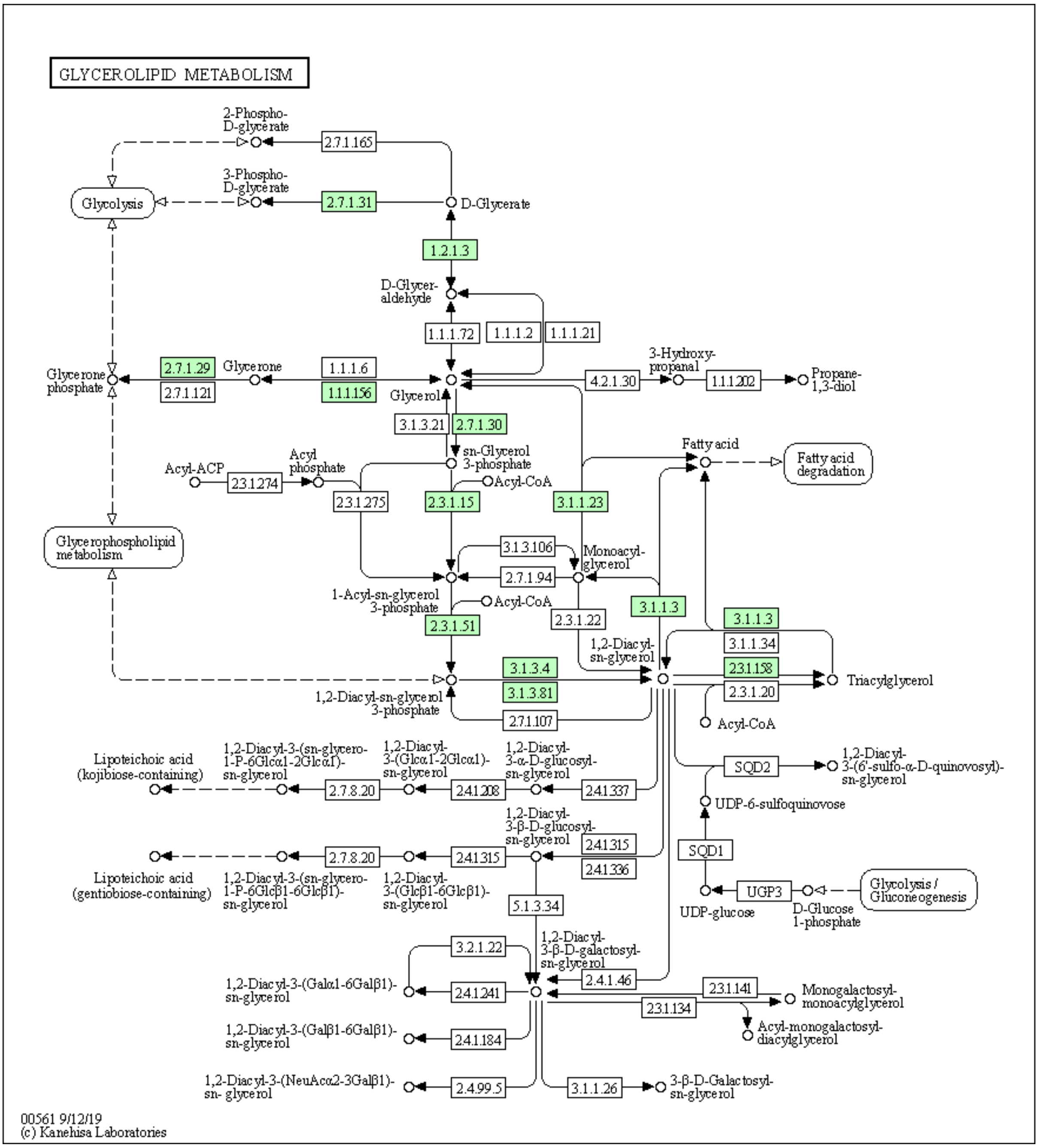

GLYCEROLIPID METABOLISM
2-Phospho-D-glycerate
3-Phospho-D-glycerate
Glycolysis
D-Glycerate
D-Glycer-aldehyde
Glycerone phosphate
Glycerone
Glycerol
3-Hydroxy-propanal
Propane-1,3-diol
sn-Glycerol 3-phosphate
Acyl-ACP
Acyl phosphate
Acyl-CoA
Fatty acid
Fatty acid degradation
Glycerophospholipid metabolism
Monoacyl-glycerol
1-Acyl-sn-glycerol 3-phosphate
1,2-Diacyl-sn-glycerol
1,2-Diacyl-sn-glycerol 3-phosphate
Triacylglycerol
Lipoteichoic acid (kojibiose-containing)
Lipoteichoic acid (gentiobiose-containing)
UDP-6-sulfoquinovose
UDP-glucose
D-Glucose 1-phosphate
Glycolysis / Gluconeogenesis
Monogalactosyl-monoacylglycerol
Acyl-monogalactosyl-diacylglycerol
3-β-D-Galactosyl-sn-glycerol
00561 9/12/19
(c) Kanehisa Laboratories

(d)

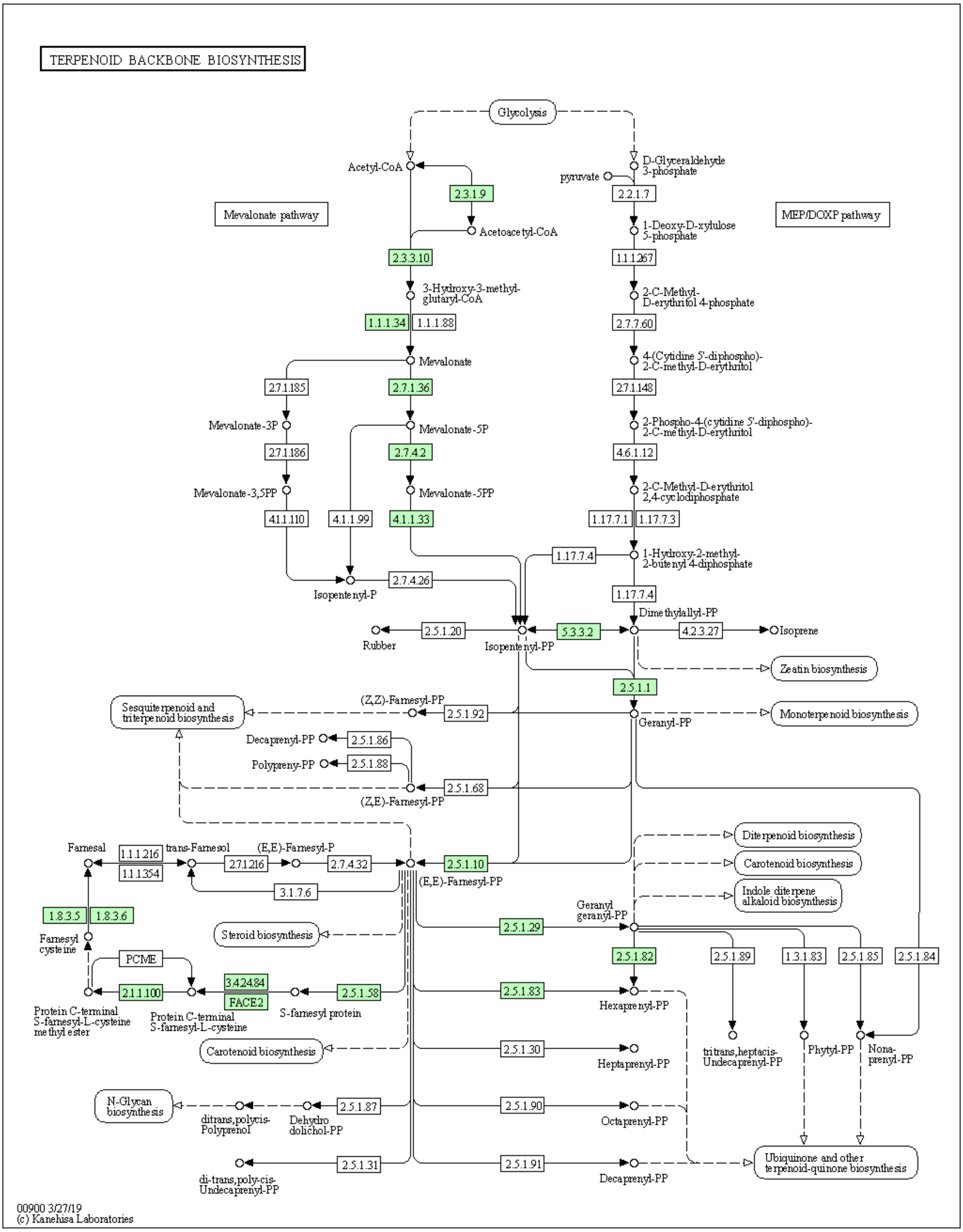

TERPENOID BACKBONE BIOSYNTHESIS
Glycolysis
Acetyl-CoA
D-Glyceraldehyde
3-phosphate
pyruvate
2.3.1.9
2.2.1.7
Mevalonate pathway
MEP/DOXP pathway
Acetoacetyl-CoA
1-Deoxy-D-xylulose
5-phosphate
2.3.3.10
1.1.1.267
3-Hydroxy-3-methyl-
glutaryl-CoA
2-C-Methyl-
D-erythritol 4-phosphate
1.1.1.34
1.1.1.88
2.7.7.60
Mevalonate
4-(Cytidine 5'-diphospho)-
2-C-methyl-D-erythritol
2.7.1.185
2.7.1.36
2.7.1.148
Mevalonate-3P
Mevalonate-5P
2-Phospho-4-(cytidine 5'-diphospho)-
2-C-methyl-D-erythritol
2.7.1.186
2.7.4.2
4.6.1.12
Mevalonate-3,5PP
Mevalonate-5PP
2-C-Methyl-D-erythritol
2,4-cyclodiphosphate
4.1.1.110
4.1.1.99
4.1.1.33
1.17.7.1
1.17.7.3
1.17.7.4
1-Hydroxy-2-methyl-
2-butenyl 4-diphosphate
Isopentenyl-P
2.7.4.26
1.17.7.4
Dimethylallyl-PP
Rubber
2.5.1.20
Isopentenyl-PP
5.3.3.2
4.2.3.27
Isoprene
Zeatin biosynthesis
2.5.1.1
Sesquiterpenoid and
triterpenoid biosynthesis
(Z,Z)-Farnesyl-PP
2.5.1.92
Geranyl-PP
Monoterpenoid biosynthesis
Decaprenyl-PP
2.5.1.86
Polypreny-PP
2.5.1.88
2.5.1.68
(Z,E)-Farnesyl-PP
Diterpenoid biosynthesis
Farnesal
1.1.1.216
1.1.1.354
trans-Farnesol
(E,E)-Farnesyl-P
2.7.1.216
2.7.4.32
2.5.1.10
Carotenoid biosynthesis
3.1.7.6
(E,E)-Farnesyl-PP
Indole diterpene
alkaloid biosynthesis
1.8.3.5
1.8.3.6
Steroid biosynthesis
Geranyl
geranyl-PP
2.5.1.29
Farnesyl
cysteine
PCME
2.5.1.82
2.5.1.89
1.3.1.83
2.5.1.85
2.5.1.84
3.4.24.84
2.1.1.100
2.5.1.58
2.5.1.83
FACE2
Hexaprenyl-PP
Protein C-terminal
S-farnesyl-L-cysteine
methyl ester
Protein C-terminal
S-farnesyl-L-cysteine
S-farnesyl protein
tritrans,heptacis-
Undecaprenyl-PP
Phytyl-PP
Nona-
prenyl-PP
Carotenoid biosynthesis
2.5.1.30
Heptaprenyl-PP
N-Glycan
biosynthesis
ditrans,polycis-
Polyprenol
Dehydro
dolichol-PP
2.5.1.87
2.5.1.90
Octaprenyl-PP
Ubiquinone and other
terpenoid-quinone biosynthesis
2.5.1.31
2.5.1.91
di-trans,poly-cis-
Undecaprenyl-PP
Decaprenyl-PP
00900 3/27/19
(c) Kanehisa Laboratories

(e)

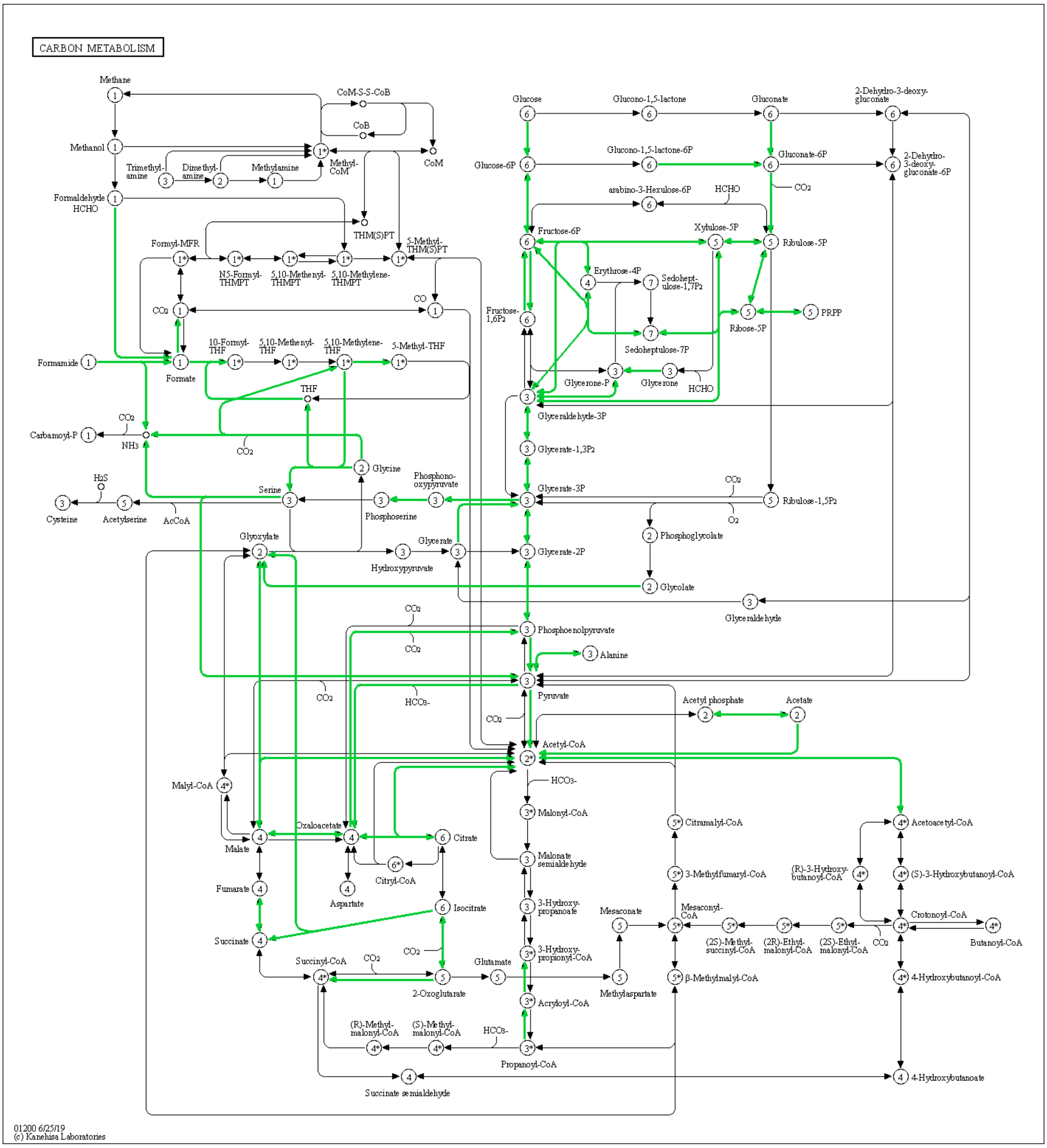

(f)

Supplementary Figure S5. Examples of lipid metabolism pathways in *Rhodotorula babjevae* DBVPG 8058 reconstructed by KEGG Mapper. The annotated transcripts with affiliated KEGG Orthology (KO) identifiers involved in each metabolic pathway are colored in green. (a) Fatty acid metabolism pathways; (b) biosynthesis of fatty acids; (c) biosynthesis of unsaturated fatty acids; (d) glycerolipid methabolism; (e) terpenoid backbone biosynthesis; (f) Carbon metabolism.

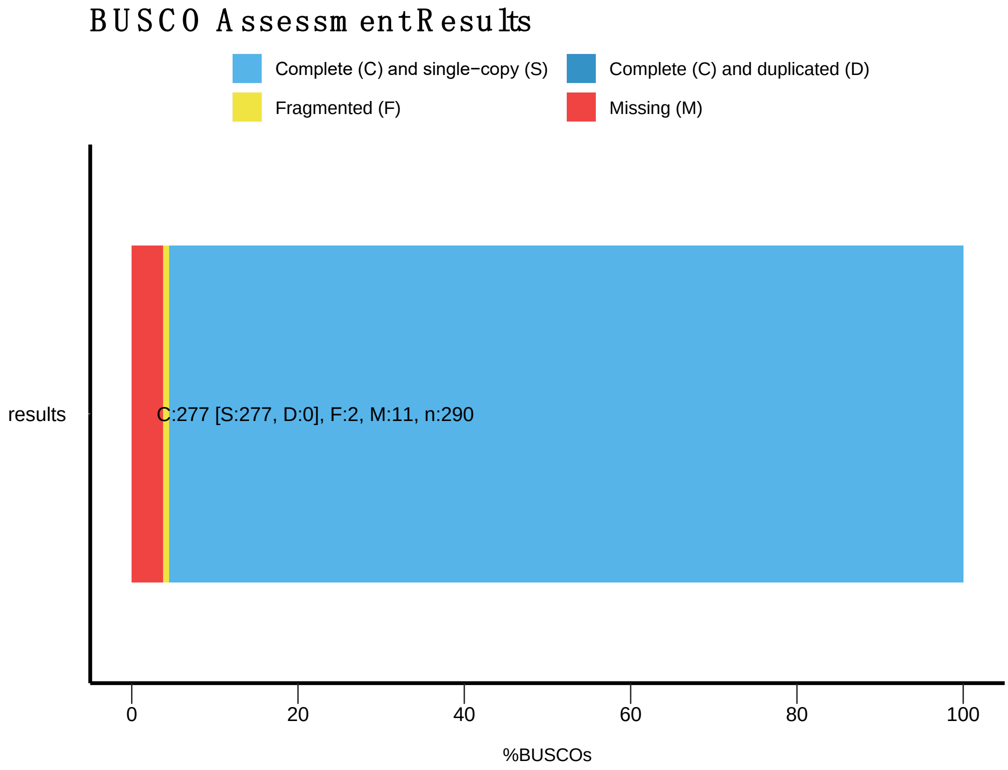

(a)

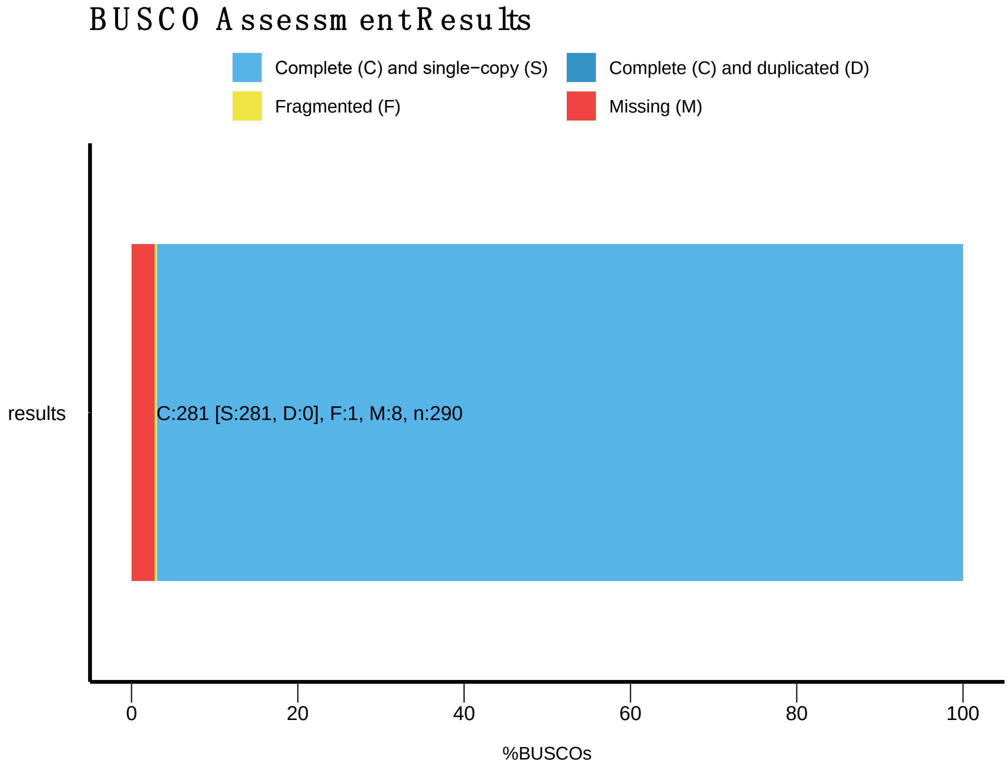

(b)

Supplementary Figure S6. Quantitative assessment of the hybrid genome assemblies and annotation completeness using Benchmarking Universal Single-Copy Orthologs (BUSCO). (a) *Rhodotorula babjevae* CBS 7808; (b) *R. babjevae* DBVPG 8058.

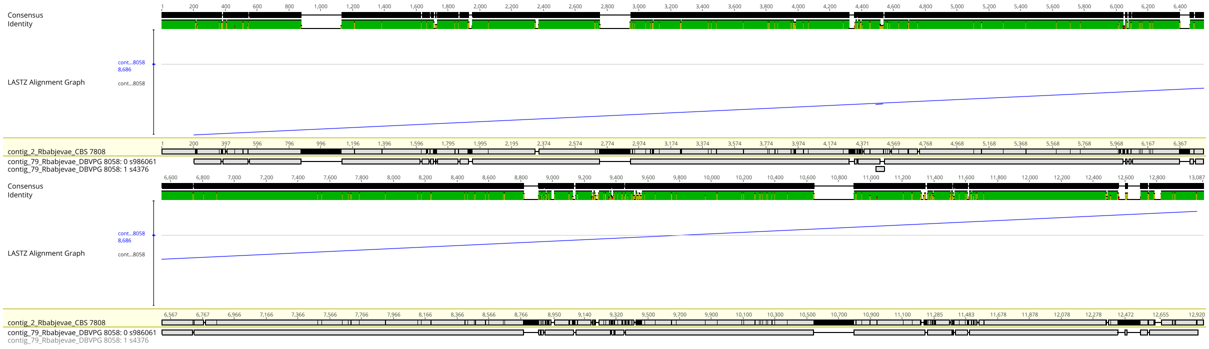

Supplementary Figure S7. LASTZ alignment of the mitochondrial genome sequences of *Rhodotorula babjevae* CBS 7808 and DBVPG 8058. The alignment was performed with the sequences after circularization. The consensus sequence is represented as a black bar. The identity bar represents pairs as green for 100% mean pairwise identity, green-brown for at least 30% and under 100%, and red for below 30%. In the bar representation of each sequence, pairs with 100% mean pairwise identity are gray and the gaps are represented either as a horizontal line in the contig that lacks the sequence or as a black bar in the contig that contains the sequence. Within the LASTZ alignment diagram, unique forward alignments are shown in blue and unique backward alignments are shown in red.

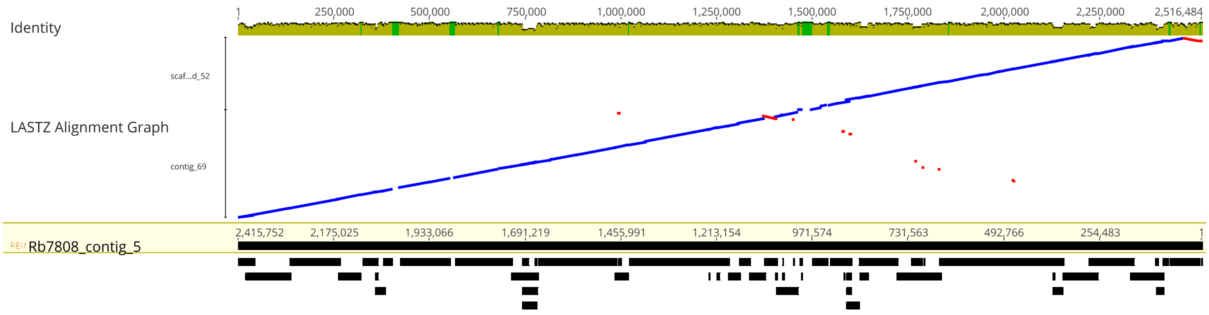
Identity
1
250,000
500,000
750,000
1,000,000
1,250,000
1,500,000
1,750,000
2,000,000
2,250,000
2,516,484
LASTZ Alignment Graph
scaf...d_52
contig_69
2,415,752
2,175,025
1,933,066
1,691,219
1,455,991
1,213,154
971,574
731,563
492,766
254,483
Rb7808_contig_5

(a)

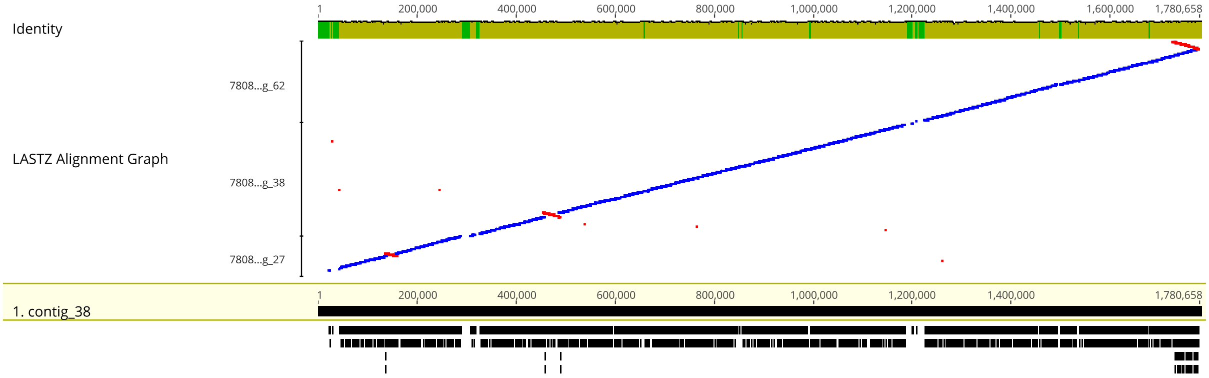
Identity
1
200,000
400,000
600,000
800,000
1,000,000
1,200,000
1,400,000
1,600,000
1,780,658
LASTZ Alignment Graph
7808...g_62
7808...g_38
7808...g_27
1. contig_38

(b)

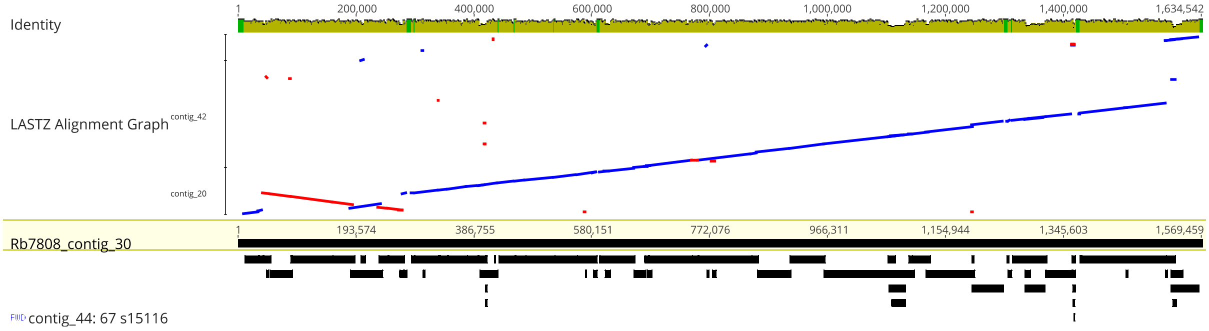
Identity
1
200,000
400,000
600,000
800,000
1,000,000
1,200,000
1,400,000
1,634,542
LASTZ Alignment Graph
contig_42
contig_20
193,574
386,755
580,151
772,076
966,311
1,154,944
1,345,603
1,569,459
Rb7808_contig_30
contig_44: 67 s15116

(c)

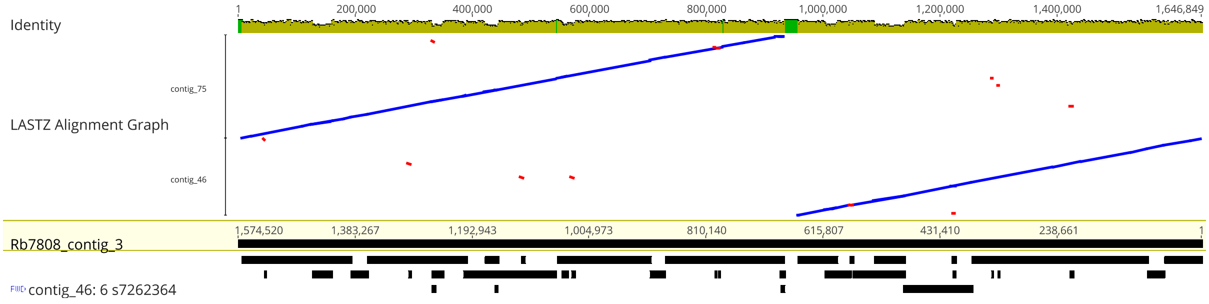
Identity
1
200,000
400,000
600,000
800,000
1,000,000
1,200,000
1,400,000
1,646,849
LASTZ Alignment Graph
contig_75
contig_46
1,574,520
1,383,267
1,192,943
1,004,973
810,140
615,807
431,410
238,661
Rb7808_contig_3
contig_46: 6 s7262364

(d)

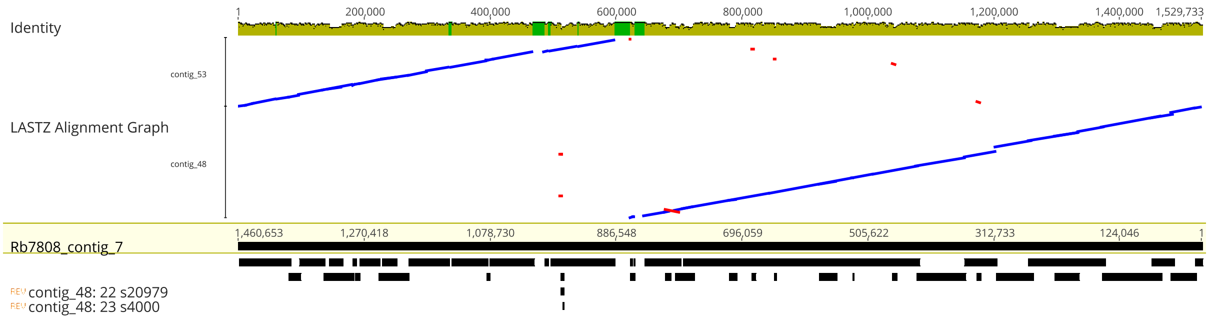

Identity
1
200,000
400,000
600,000
800,000
1,000,000
1,200,000
1,400,000
1,529,733
LASTZ Alignment Graph
contig_53
contig_48
Rb7808_contig_7
1,460,653
1,270,418
1,078,730
886,548
696,059
505,622
312,733
124,046
1
contig_48: 22 s20979
contig_48: 23 s4000

(e)

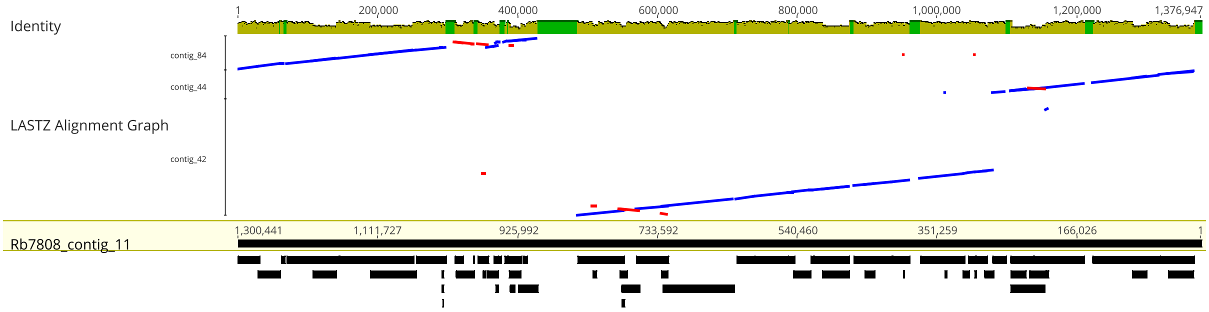

Identity
1
200,000
400,000
600,000
800,000
1,000,000
1,200,000
1,376,947
LASTZ Alignment Graph
contig_84
contig_44
contig_42
Rb7808_contig_11
1,300,441
1,111,727
925,992
733,592
540,460
351,259
166,026
1

(f)

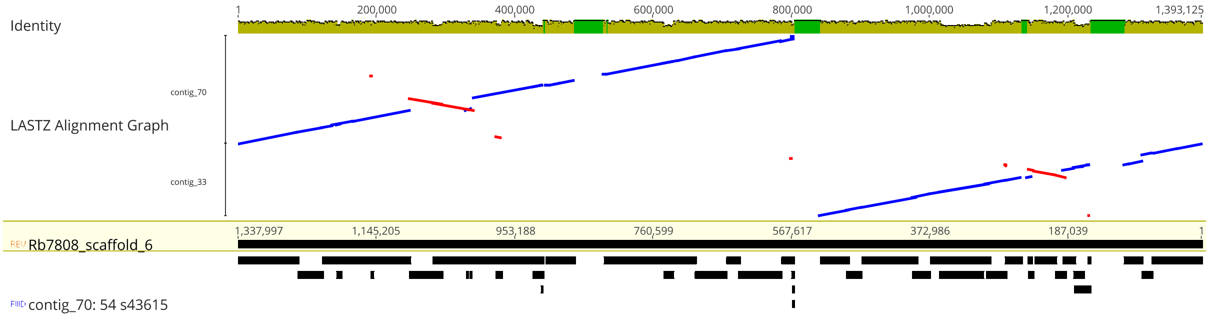

Identity
1
200,000
400,000
600,000
800,000
1,000,000
1,200,000
1,393,125
LASTZ Alignment Graph
contig_70
contig_33
Rb7808_scaffold_6
1,337,997
1,145,205
953,188
760,599
567,617
372,986
187,039
1
contig_70: 54 s43615

(g)

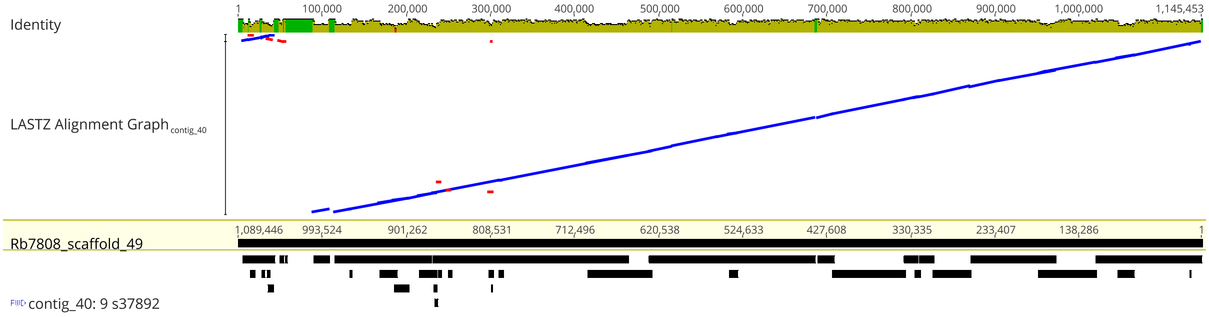

Identity
1
100,000
200,000
300,000
400,000
500,000
600,000
700,000
800,000
900,000
1,000,000
1,145,453
LASTZ Alignment Graph
contig_40
Rb7808_scaffold_49
1,089,446
993,524
901,262
808,531
712,496
620,538
524,633
427,608
330,335
233,407
138,286
1
contig_40: 9 s37892

(h)

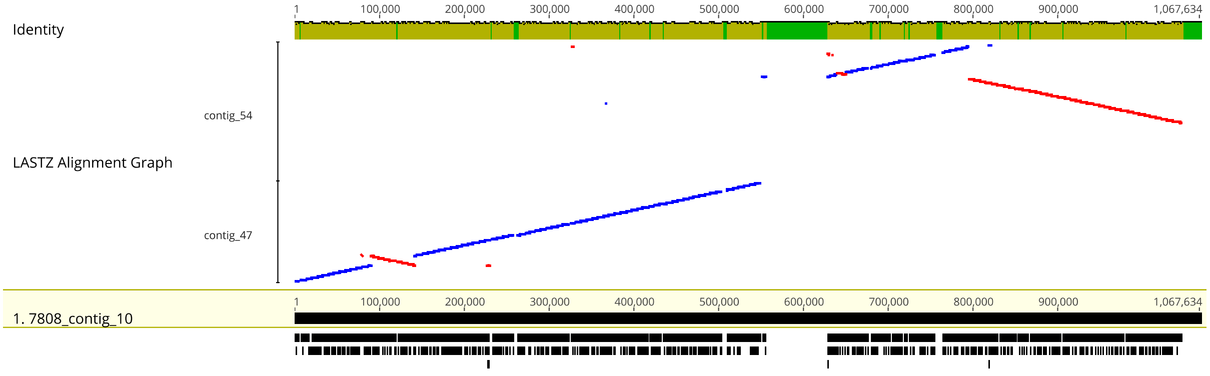
Identity
LASTZ Alignment Graph
contig_54
contig_47
1. 7808_contig_10
1
100,000
200,000
300,000
400,000
500,000
600,000
700,000
800,000
900,000
1,067,634

(i)

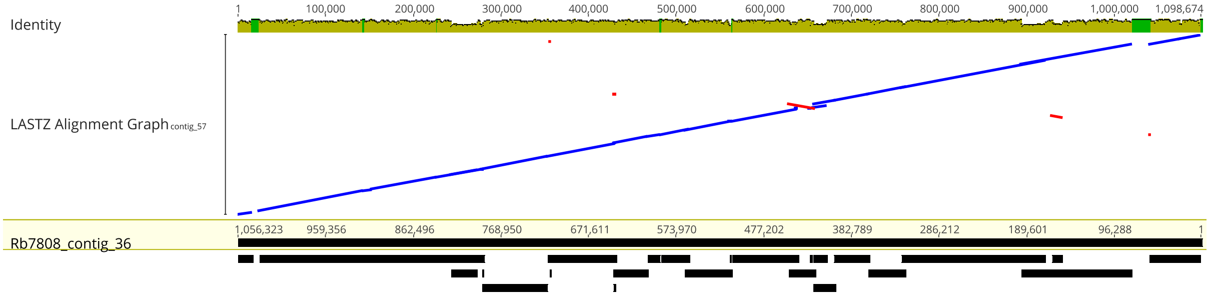
Identity
LASTZ Alignment Graph
contig_57
Rb7808_contig_36
1
100,000
200,000
300,000
400,000
500,000
600,000
700,000
800,000
900,000
1,000,000
1,098,674
1,056,323
959,356
862,496
768,950
671,611
573,970
477,202
382,789
286,212
189,601
96,288
1

(j)

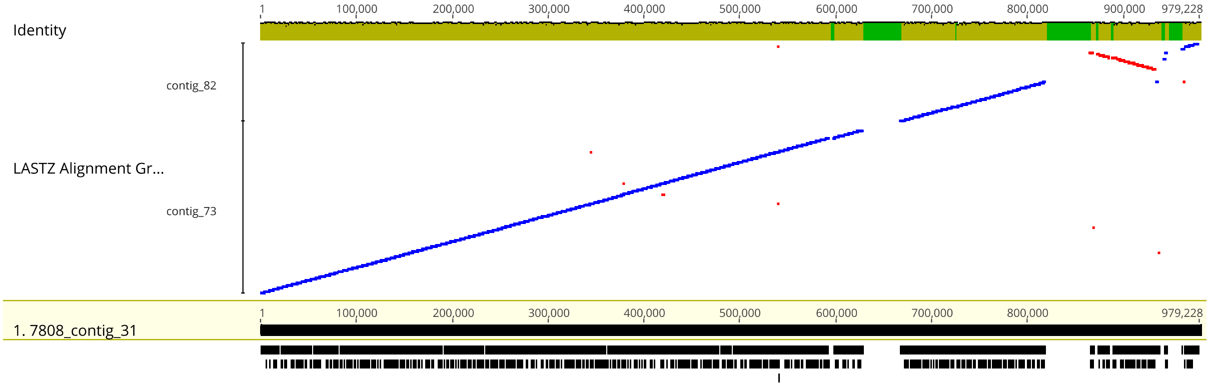
Identity
LASTZ Alignment Gr...
contig_82
contig_73
1. 7808_contig_31
1
100,000
200,000
300,000
400,000
500,000
600,000
700,000
800,000
900,000
979,228

(k)

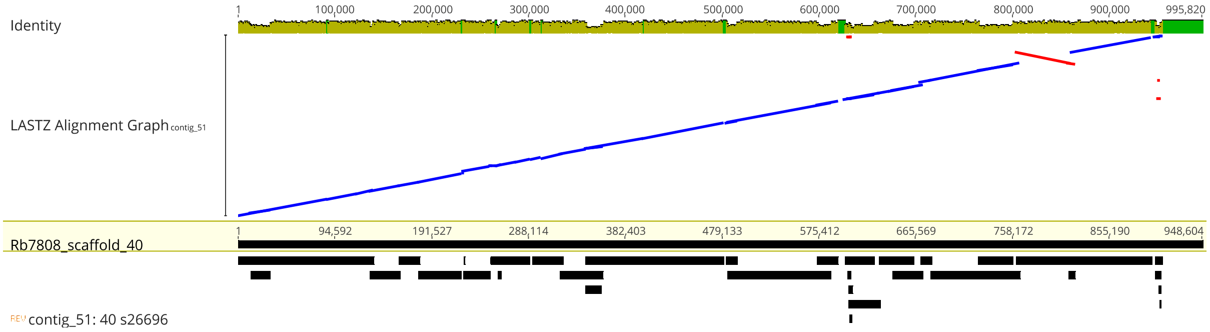
Identity
LASTZ Alignment Graph
contig_51
Rb7808_scaffold_40
1
100,000
200,000
300,000
400,000
500,000
600,000
700,000
800,000
900,000
995,820
94,592
191,527
288,114
382,403
479,133
575,412
665,569
758,172
855,190
948,604
contig_51: 40 s26696

(l)

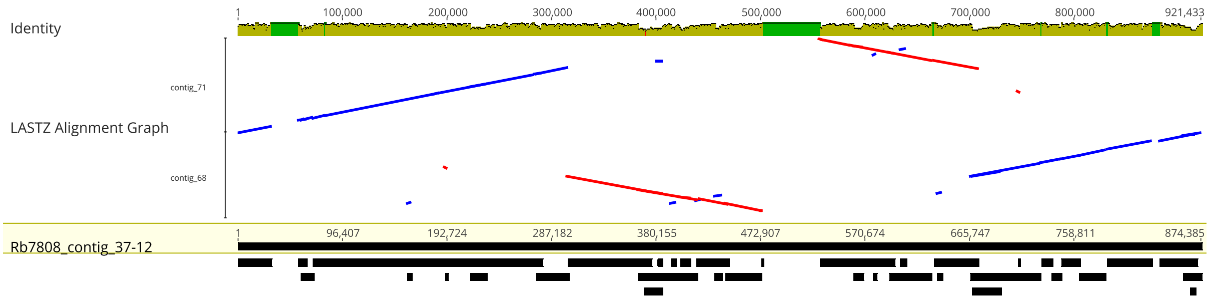
Identity
LASTZ Alignment Graph
contig_71
contig_68
1 100,000 200,000 300,000 400,000 500,000 600,000 700,000 800,000 921,433
Rb7808_contig_37-12
1 96,407 192,724 287,182 380,155 472,907 570,674 665,747 758,811 874,385

(m)

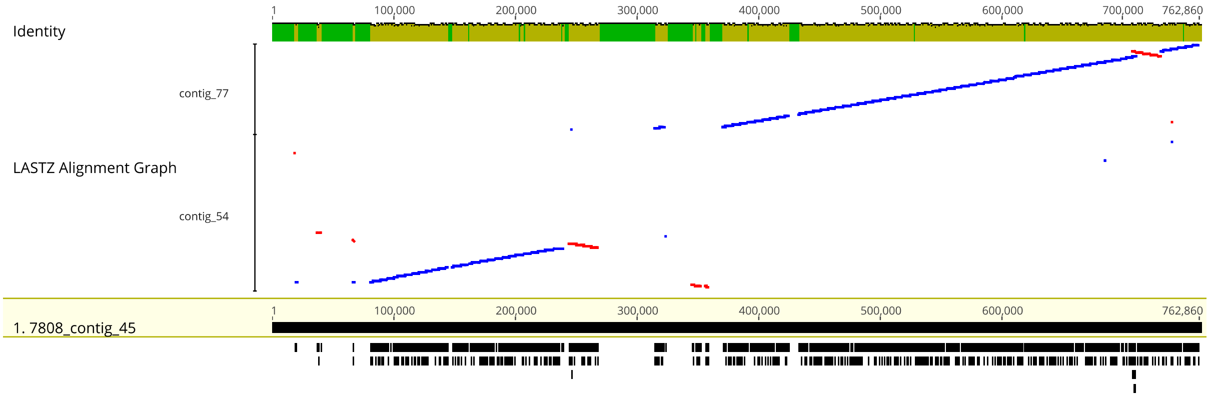
Identity
LASTZ Alignment Graph
contig_77
contig_54
1 100,000 200,000 300,000 400,000 500,000 600,000 700,000 762,860
1. 7808_contig_45
1 100,000 200,000 300,000 400,000 500,000 600,000 762,860

(n)

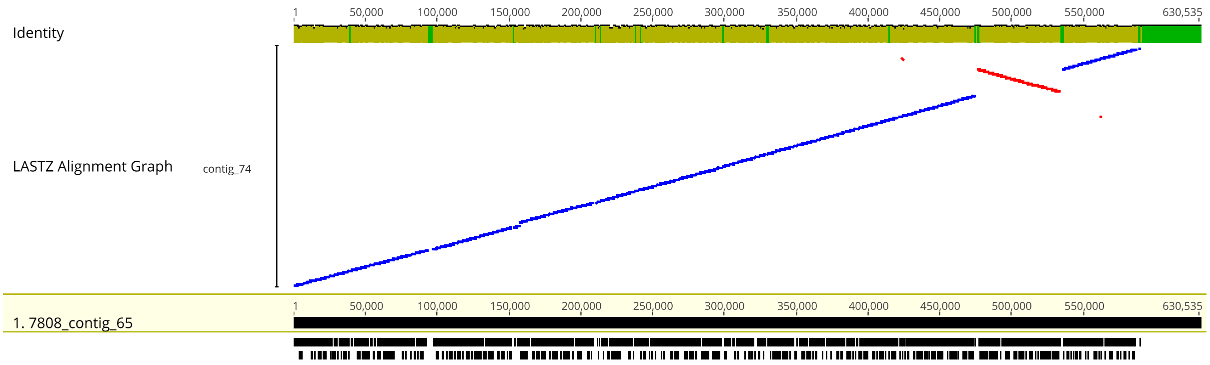
Identity
LASTZ Alignment Graph
contig_74
1 50,000 100,000 150,000 200,000 250,000 300,000 350,000 400,000 450,000 500,000 550,000 630,535
1. 7808_contig_65
1 50,000 100,000 150,000 200,000 250,000 300,000 350,000 400,000 450,000 500,000 550,000 630,535

(o)

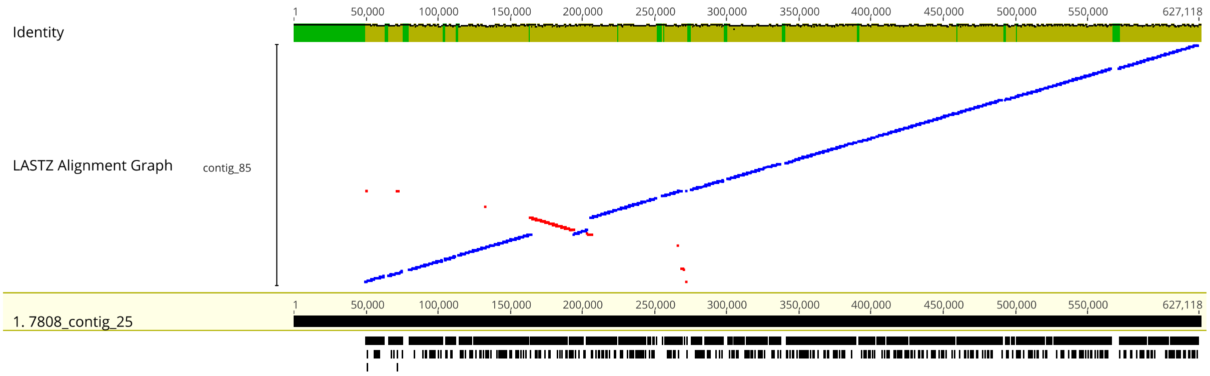
Identity
LASTZ Alignment Graph
contig_85
1 50,000 100,000 150,000 200,000 250,000 300,000 350,000 400,000 450,000 500,000 550,000 627,118
1. 7808_contig_25
1 50,000 100,000 150,000 200,000 250,000 300,000 350,000 400,000 450,000 500,000 550,000 627,118

(p)

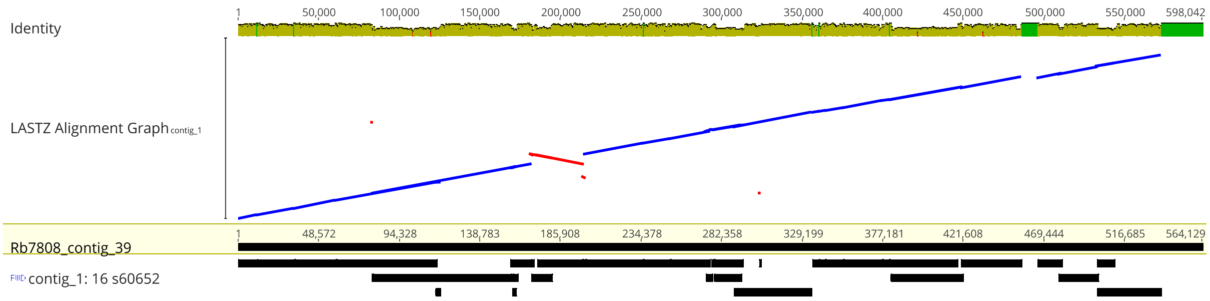
Identity
LASTZ Alignment Graph contig_1
1 50,000 100,000 150,000 200,000 250,000 300,000 350,000 400,000 450,000 500,000 550,000 598,042
Rb7808_contig_39
1 48,572 94,328 138,783 185,908 234,378 282,358 329,199 377,181 421,608 469,444 516,685 564,129
contig_1: 16 s60652

(q)

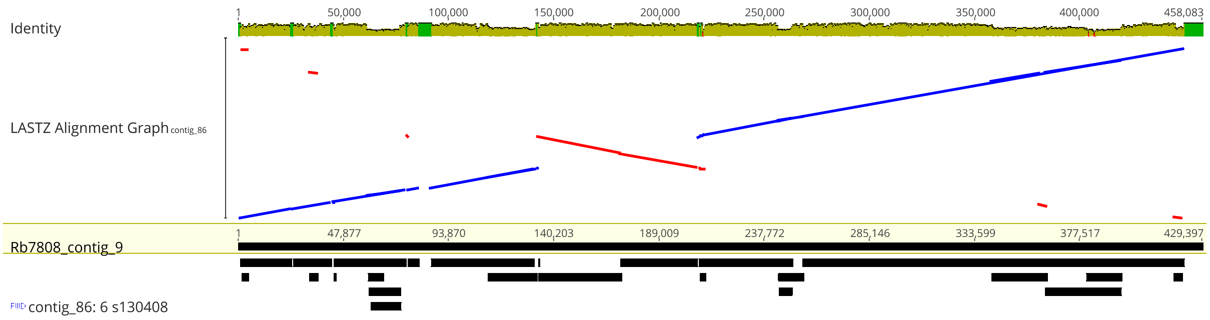
Identity
LASTZ Alignment Graph contig_86
1 50,000 100,000 150,000 200,000 250,000 300,000 350,000 400,000 458,083
Rb7808_contig_9
1 47,877 93,870 140,203 189,009 237,772 285,146 333,599 377,517 429,397
contig_86: 6 s130408

(r)

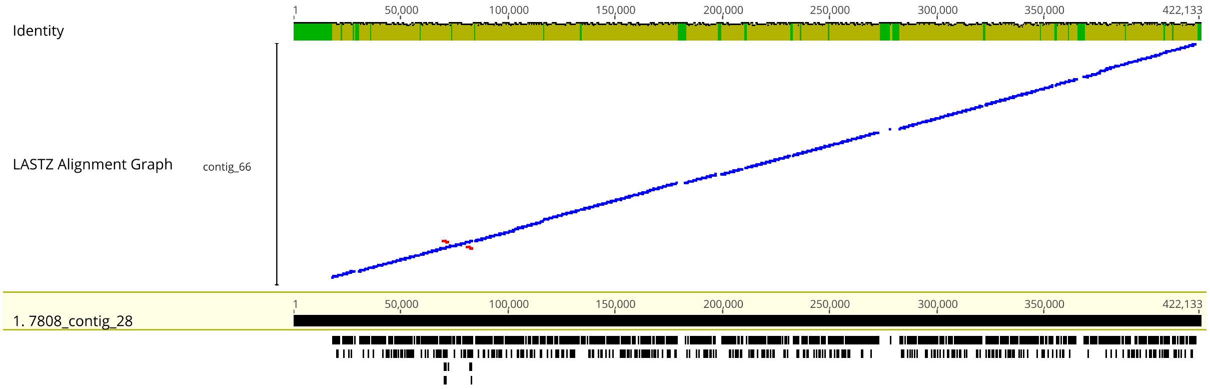
Identity
LASTZ Alignment Graph
contig_66
1 50,000 100,000 150,000 200,000 250,000 300,000 350,000 422,133
1. 7808_contig_28
1 50,000 100,000 150,000 200,000 250,000 300,000 350,000 422,133

(s)

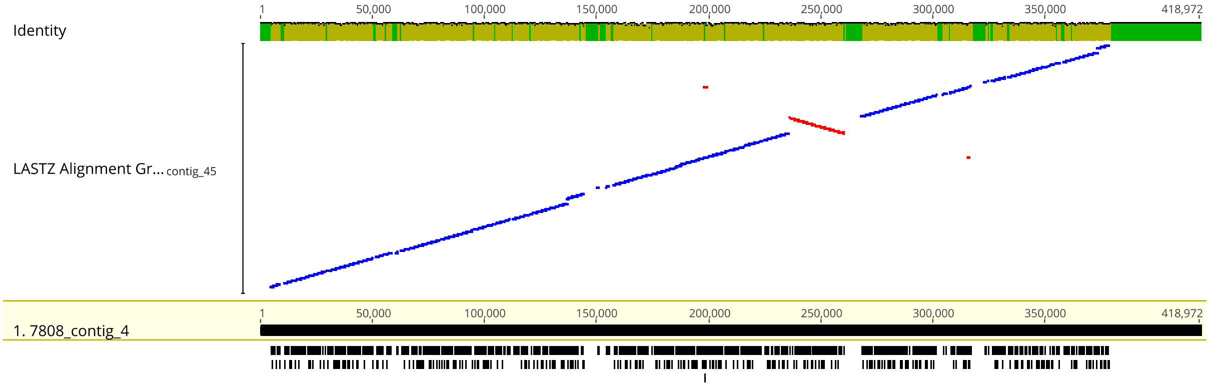
Identity
LASTZ Alignment Gr... contig_45
1 50,000 100,000 150,000 200,000 250,000 300,000 350,000 418,972
1. 7808_contig_4
1 50,000 100,000 150,000 200,000 250,000 300,000 350,000 418,972

(t)

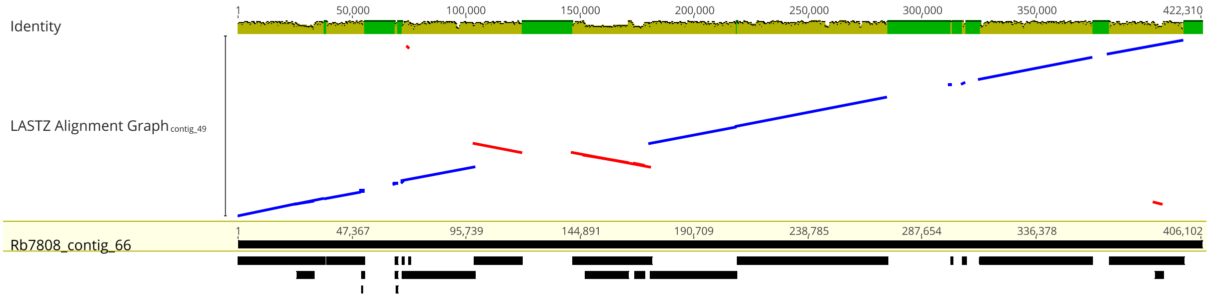

(u)

Supplementary Figure S8. LASTZ alignment of contigs with assigned homology of *Rhodotorula babjevae* CBS 7808 and DBVPG 8058 representing putative chromosomes. *R. babjevae* putative chromosomes: (a) 1, (b) 2, (c) 3, (d) 4, (e) 5, (f) 6, (g) 7, (h) 8, (i) 9, (j) 10, (k) 11, (l) 12, (m) 13, (n) 14, (o) 15, (p) 16, (q) 17, (r) 18, (s) 19, (t) 20, (u) 21. The identity bar represents pairs as green for 100% mean pairwise identity, green-brown for at least 30% and under 100%, and red for below 30%. In the bar representation of each sequence, pairs with 100% mean pairwise identity are gray and the gaps are represented either as a horizontal line in the contig that lacks the sequence or as a black bar in the contig that contains the sequence. Within the LASTZ alignment diagram, unique forward alignments are shown in blue and unique backward alignments are shown in red.
